# PPE57 Cooperates with MmpL3 to Mediate Bacterial Lipid Transport and Promote Persistent Infection of *Mycobacterium tuberculosis*

**DOI:** 10.3390/microorganisms14091912

**Published:** 2026-08-28

**Authors:** Shufeng Weng, Qingchun Li, Yamin Zhao, Taiyue Lin, Zihan Wang, Mingrui Zhu, Miaochengyue Xin, Ying Xu

**Affiliations:** 1 State Key Laboratory of Genetic Engineering, School of Life Science, Institute of Infection and Health, National Medical Center for Infectious Diseases, Fudan University, Shanghai 200438, China; 2 Shanghai Sci-Tech Inno Center for Infection & Immunity, Shanghai 200052, China; 3 MOE Engineering Research Center of Gene Technology, Shanghai Engineering Research Center of Industrial Microorganism, Fudan University, Shanghai 200433, China

**Keywords:** *Mycobacterium tuberculosis*, PPE57, mycolic acid, MmpL3, cholesterol

## Abstract

*Mycobacterium tuberculosis* (*M. tb*) possesses a unique, lipid-rich cell envelope that is critical for virulence, drug resistance, and persistence. Here, we identify the PPE family protein PPE57 as a key regulator of mycolic acid transport and host lipid exploitation. PPE57 physically interacts with the essential lipid transporter MmpL3, promoting trehalose monomycolate (TMM) translocation, enhancing trehalose dimycolate (TDM) synthesis, and increasing cell wall lipid content. Site-directed mutagenesis identified G175 as a critical residue for PPE57-MmpL3 binding. Deletion of PPE57 reduces cell wall thickness, alters lipid composition, and impairs biofilm formation. Mechanistically, PPE57 facilitates bacterial cholesterol acquisition, and during infection, activates the host PPAR-γ pathway in macrophages, leading to enhanced cholesterol uptake, lipid droplet accumulation, and increased intracellular triglyceride and cholesteryl ester levels. These changes provide a nutrient-rich niche that promotes bacterial survival and persistence. In a mouse model of infection, PPE57 deficiency results in reduced bacterial burden, milder lung pathology, and diminished lipid droplet-positive cell accumulation in lung tissues. These findings establish PPE57 as an essential accessory component of the MmpL3 lipid transport system, bridging mycobacterial cell wall assembly with host nutrient exploitation during persistent infection.

## 1. Introduction

*Mycobacterium tuberculosis* (*M. tb*) is one of the most widespread intracellular pathogens globally, infecting approximately one-quarter of the world’s population and causing an estimated 1.3 million deaths annually [1]. The remarkable pathogenicity of *M. tb* is largely attributed to its unique cell envelope, particularly the outer membrane (OM), which acts as a formidable barrier against host defenses and antibiotics. This OM is an impermeable lipid bilayer composed primarily of mycolic acids and associated free lipids [2,3,4]. The outer membrane lipids are essential for the growth of M. tb, accounting for about 40% of the bacterial dry weight, and are crucial for maintaining the envelope permeability, plasma membrane integrity, and bacterial replication [5]. Beyond their structural functions, mycobacterial lipids also serve as key virulence determinants, actively shaping host–pathogen interactions. Accumulating evidence indicates that these lipids serve as effector molecules that reprogram host cell metabolism and elicit immune responses [6,7]. Thus, pathways involved in lipid biosynthesis and transport have emerged as important targets for anti-tuberculosis drug development.

The outer membrane of *M. tb* exhibits unique characteristics—long-chain branched fatty acids are enveloped by abundant mycolic acids (MAs), forming a bilayer with significantly reduced fluidity and permeability [8,9]. These MAs exist in the form of trehalose monomycolates (TMMs), trehalose dimycolates (TDMs), and mycolic acids covalently linked to the arabinogalactan (AG) polysaccharide, which in turn is connected to peptidoglycan, collectively known as the mAGP complex [10,11]. TMM is synthesized on the inner membrane (IM) as a precursor of MA through a highly conserved and well-characterized pathway, which is the target of the first-line anti-tuberculosis drug isoniazid [9,12]. MmpL3 mediates the translocation of TMM across the cytoplasm and IM [13], yet the subsequent steps remain largely uncharacterized. Whether the delivery of TMM to the Ag85 complex requires an unidentified transport system remains unknown [14].

Emerging evidence suggests that the PE/PPE protein families, which account for approximately 10% of the *M. tb* coding genome, may serve as such accessory components in mycobacterial lipid metabolism and transport [15,16,17]. Several family members, including PPE71 and PE11, have been shown to influence cell wall lipid content [18,19], and computational analyses have predicted similar roles for PE_PGRS10 and PPE21 [20]. Moreover, certain PE/PPE proteins localize to the outermost layers of the cell envelope and have been implicated in transporting diverse molecules across the mycobacterial envelope—including iron (PPE36/PPE62/PPE64), calcium (PPE37), and magnesium (PPE31), among others [21,22,23]. Notably, PPE51 and PE19 are proposed to form a channel that facilitates transmembrane lipid movement, although the underlying mechanisms remain elusive [15,24]. Given their spatial distribution and functional links to lipid metabolism, we hypothesized that additional PE/PPE family members may directly participate in mycolic acid transport and outer membrane assembly.

In this study, we identify the PPE family protein PPE57 as a critical regulator of this process. We demonstrate that PPE57 physically interacts with MmpL3, thereby promoting TMM transport, TDM synthesis, and cell wall lipid content in the mycobacterial cell wall. Independent studies, including our own and those by Zhu et al., implicated PPE57 in mycobacterial physiology, showing that it affects membrane integrity and function in *M. smegmatis* and avirulent *M. tuberculosis* H37Ra, contributing to stress tolerance, drug resistance, and virulence [25,26]. However, the underlying molecular mechanisms remained unexplored, particularly in the context of virulent *M. tb*. In a virulent strain, PPE57 knockout reveals that PPE57 facilitates host cholesterol acquisition via modulating mycolic acid transport and outer membrane composition, enhancing bacterial intracellular survival and persistent infection. Thus, PPE57 links mycobacterial lipid transport to host nutrient exploitation.

## 2. Materials and Methods

### 2.1. Ethics Statement

All mice were housed under specific pathogen free conditions in the animal center, School of Life Sciences, Fudan University. All experimental procedures were in accordance with the guide for the care and use of laboratory animals of the National Institutes of Health and approved by the animal care and use ethics committee of Fudan University (animal ethics license number: 2023-HSYY-019). According to the biosafety rules of Fudan University, *M. tb* related cell experiments and mouse infection were carried out in the ABSL-3 laboratory.

### 2.2. Bacterial Strains and Cell Lines

BCG (Pasteur strain), *M. tuberculosis H37Ra* (ATCC 25177), and *M. tuberculosis H37Rv* (ATCC 27294) strains were grown at 37 °C in Middlebrook 7H9 broth (Cat#271310, Difco, Sparks, MD, USA) supplemented with 0.2% glycerol, 0.05% Tween 80, and 10% oleic acid-albumin-dextrose-catalase (OADC) or on Middlebrook 7H10 agar (Cat#262710, Difco, USA) supplemented with 0.5% glycerol and 10% OADC. Sauton’s medium was used for culture filtrate analysis. *E. coli* DH5α and BL21 were grown in LB medium for genetic manipulations or protein overexpression. RAW264.7 cells (Cat#TIB-71, ATCC, Manassas, VA, USA) were cultured in DMEM and THP-1 cells (Cat#TIB-202, ATCC, USA) were cultured in RPMI-1640 supplemented with 10% fetal bovine serum (FBS), penicillin (100 U/mL), and streptomycin (100 µg/mL) and maintained at 37 °C in a humidified incubator with 5% CO_2_. Six-week-old female C57BL/6J mice were purchased from Charles River Laboratories (Beijing, China).

The *pMV261-ppe57* construct was generated by enzymatic ligation (Table S2). The sequences encoding PPE57 was amplified from *M. tb H37Rv* by PCR. The amplified fragment was digested with restriction endonuclease and ligated into the linearized plasmid. The recombinant plasmid was sequenced by Shanghai Sangon (Shanghai, China), and the sequence was confirmed by BLAST analysis (https://blast.ncbi.nlm.nih.gov/Blast.cgi, 23 July 2023). The successfully constructed plasmid was electro transformed into the BCG recipient strain, and the empty vector pMV261 was also electroporated to generate the BCG-Vec strain.

### 2.3. Mouse Infection Experiment

Mice were randomly assigned to groups using a random number table, with cage positions and processing orders balanced across groups to minimize positional and batch effects. Six-week-old female C57BL/6J mice (Charles River, Beijing, China) were acclimatized for one week in the ABSL-3 laboratory. For aerosol infection, mice were exposed to approximately 100 CFU of the indicated *M. tb* strains using an aerosol inhalation device. No anesthesia was required for the aerosol exposure procedure, as the mice were physically restrained without causing distress, and the procedure is non-invasive.

At 3, 7, and 28 days post-infection, six mice from each group (*H37Rv*, Δ*PPE57*, Δ*PPE57::PPE57, H37Rv::PPE57*) were euthanized for analysis. For euthanasia, deep anesthesia was first induced by intraperitoneal injection of tribromoethanol (Avertin; Cat#M2940, Aibei, Guangzhou, China) at a dose of 270 mg/kg body weight. Anesthesia depth was carefully monitored and confirmed by the complete loss of pedal withdrawal reflex and corneal reflex. To ensure death, all mice were subsequently subjected to cervical dislocation as a secondary physical method immediately after confirmation of deep anesthesia. All efforts were made to minimize animal suffering in accordance with the institutional guidelines.

After surface disinfection with alcohol spray, the mice were dissected. The upper lobe of the right lung and the spleen were removed, washed with PBS, weighed, ground, and diluted for CFU counting. One hundred microliters of the homogenate supernatant was centrifuged at 12,000 rpm for 10 min, and the supernatant was collected for cytokine detection. The upper lobe of the left lung was fixed with paraformaldehyde solution for pathological analysis. The lower lobe of the left lung was stored in a −80 °C freezer for lipid analysis.

### 2.4. Mutant Construction

The *ppe57* knockout mutant was created by specialized phage transduction. Briefly, the right (756 bp) and left arms (702 bp) of the *ppe57* gene were PCR amplified using flanking primers. The arms were cloned into p0004S vector and sequence verified (Table S1). Positive clones were further transformed into *E. coli* HB101 cells together with shuttle phasmid phAE159. High-titer phages were prepared and infected with *M. tb* H37Rv accordingly to previously described methods [27]. The knock-out colonies were screened by PCR.

To construct the complemented and overexpressing strain, Δ*PPE57* and H37Rv were respectively transformed with the complementing plasmid *pMV361-ppe57*, which carries the *ppe57* gene under the control of the HSP60 promoter. In brief, *pMV361-ppe5*7 was electroporated into the electro-competent cells of Δ*PPE57* or WT as described previously [28]. The knockout, complemented, and overexpressing strains were confirmed by RT-qPCR and Western blot.

### 2.5. Colony Morphology and Pellicular Biofilm Formation

The four strains were grown in liquid medium to an OD600 of 0.5, and 100 µL of each culture was spread on 7H10 agar plates supplemented with 1% glycerol, 10% OADC, 0.05% Tween-80, and corresponding antibiotics. Plates were incubated at 37 °C for 4–6 weeks. The high-resolution microphotography of the colonies was carried out by a Nikon Camera. The strains were grown to mid-log phase and diluted. For pellicle formation, all cultures were grown in Sauton’s medium at 37 °C without shaking and photographed at 2-day intervals.

### 2.6. Observation by Transmission Electron Microscopy

Log-phase bacteria were collected and washed with ddH_2_O, then fixed with 2.5% glutaraldehyde solution (Cat#P1126, Solarbio, Beijing, China). A 2.5 µL aliquot of the bacterial suspension was placed onto a 300-mesh carbon grid that had undergone glow discharge. Excess liquid was gently blotted with filter paper. Then, 2.5 µL of uranyl acetate stain was added, and excess liquid was again gently blotted. The samples were observed under transmission electron microscope. The thickness of the bacterial cell wall was measured and recorded.

### 2.7. Minimum Inhibitory Concentration Detection

The minimum inhibitory concentration (MIC) against Mycobacterium tuberculosis was determined by the broth microdilution assay. Briefly, twice the serial dilutions of drugs (all purchased from Sangon, Shanghai, China) were formulated in 7H9-OADC and dispensed into 96-well microplates. A standardized bacterial inoculum prepared from freshly cultured colonies was added to each well. The plates were incubated at 37 °C, and bacterial growth was assessed using a 2 µg/mL resazurin chromogenic test at 20 days of culture. MIC was recorded as the lowest concentration that completely inhibited visible growth. Each assay was performed in duplicate, and growth and sterile controls were included in each plate.

### 2.8. Mycobacterial Lipid Extraction and TMM/TDM TLC Analysis

Log-phase bacteria were collected separately, and the residual culture medium was removed. Then, 10 mL of the extraction solvent (chloroform:methanol:water = 65:25:4) was added to the bacterial pellet. After shaking and mixing thoroughly, the mixture was extracted overnight. The extract was centrifuged at 4000 rpm for 10 min to separate the layers, and the organic phase was collected. The total polar lipids were obtained by rotary evaporation at 50 °C. When used, the lipids were redissolved in chloroform.

A starting line and sample application positions were marked with a pencil on the TLC plate (Cat#A524752, Sangon, China). The starting line was 1 cm from the bottom of the TLC plate, and samples were spaced 1 cm apart. A total of 200 µg of total lipids was applied to the sample spots using a capillary tube. The developing solvent (chloroform:methanol:ammonia = 80:20:2) was added to the developing chamber 20 min in advance and mixed thoroughly. The height of the developing solvent should not exceed 1 cm. When the developing solvent migrated to about 1 cm from the top of the TLC plate, the chromatography was stopped. The TLC plate was removed and air dried for 3–5 min at room temperature. The plate was sprayed twice with a 5% phosphomolybdic acid solution (Cat#A600709, Sangon, China) and then developed by heating with a hot air gun at 120 °C for 3–5 min. The results were photographed and subjected to grayscale semiquantitative analysis using ImageJ software (version 1.47).

### 2.9. M. tb Lipidome Analysis

Lipids were extracted using the methyl tert butyl ether (MTBE) method. Briefly, samples were first spiked with an appropriate amount of internal lipid standards and then homogenized with 200 µL water and 240 µL methanol. After that, 800 µL of MTBE was added, and the mixture underwent ultrasound for 20 min at 4 °C followed by sitting still for 30 min at room temperature. The solution was centrifuged at 14,000× *g* for 15 min at 10 °C, and the upper organic solvent layer was obtained and dried under nitrogen. Reverse phase chromatography was selected for LC separation using the CSH C18 column (1.7 μm, 2.1 mm× 100 mm, Waters). The lipid extracts were re-dissolved in 200 µL 90% isopropanol/acetonitrile, centrifuged at 14,000× *g* for 15 min, and finally 3 µL of sample was injected. Solvent A was acetonitrile–water (6:4, *v*/*v*) with 0.1% formic acid and 0.1 Mm ammonium formate and solvent B was acetonitrile–isopropanol (1:9, *v*/*v*) with 0.1% formic acid and 0.1 Mm ammonium formate. The initial mobile phase was 30% solvent B at a flow rate of 300 μL/min. This was held for 2 min, and then linearly increased to 100% solvent B in 23 min, followed by equilibrating at 5% solvent B for 10 min. Mass spectra were acquired by Q-Exactive Plus in positive and negative mode, respectively. ESI parameters were optimized and preset for all measurements as follows: Source temperature, 300 °C; capillary temp, 350 °C, the ion spray voltage was set at 3000 V, S-Lens RF level was set at 50%, and the scan range of the instrument was set at *m*/*z* 200–1800. “Lipid Search” is a search engine for the identification of lipid species based on MS/MS math. Lipid Search contains more than 30 lipid classes and more than 1,500,000 fragment ions in the database. Both mass tolerance for the precursor and fragment were set to 5 ppm.

### 2.10. MST Detection

The affinities were measured using MicroScale Thermophoresis (Monolith NT.115, Nanotemper, Munich, Germany) following the manufacturer’s instruction. Briefly, purified MmpL3 protein was labeled with the fluorescent dye Red-NHS using the Monolith NT Protein Labeling Kit (#MO-L011, Nanotemper, Germany). A fixed concentration of NHS-Red labeled MmpL3 (20 nM) was incubated with serially diluted PPE57 at a 2-fold dilution series (16 points) in PBS-T buffer, (0.05%), with the lowest concentration at 5 nM and the highest at approximately 160 µM. Five minutes later, the samples were loaded into glass capillaries (Cat#MO-K002, Nanotemper, Germany), and affinity measurements were performed at 37 °C. Dose–response curves of PPE57 were acquired, and the Kd values were calculated by the MO.Affinity software (Version 2.6.3). Uncoupled NHS-Red dye was used as a negative control.

### 2.11. Molecular Docking and Molecular Dynamics Simulation

#### 2.11.1. Molecular Docking

The structures of MmpL3 (PDB: 7NVH) and PPE57 (AlphaFold DB: AF-Q50703-F1) were submitted to the HDOCK server with all parameters set to default. To ensure the comprehensiveness of the selected models, model_1 was chosen. LigPlot^+^ 2.2.4 was used to identify the functional residues involved in forming hydrogen bonds, salt bridges, or hydrophobic interactions. The protein–protein docking conformation was visualized using PyMol 2.2.0. In LigPlot^+^ 2.2.4, MmpL3 was labeled as chain A, and PPE57 was labeled as chain B. In PyMol, MmpL3 was represented as a magenta cartoon model, and PPE57 was shown as a cyan cartoon model. Their binding sites were displayed as pink stick structures. For close up views of the binding interface, the interacting residues were shown as sticks colored according to the parent protein. The binding energy and dissociation constant of the complex were calculated using Prodigy (https://wenmr.science.uu.nl 21 February 2023).

#### 2.11.2. Molecular Dynamics Simulation

A 500 ns MD simulation was performed using Desmond 2021.1 to optimize the conformation and study the binding mode. The simulation system was prepared by applying the OPLS4 force field to the complex. An orthogonal box with a 10 Å boundary around the complex was generated, which was then filled with SPC water molecules. The system was neutralized by adding 1.5 M NaCl and additional Na^+^ and Cl^−^ ions (other parameters were set to default).

### 2.12. Protein Expression and Purification

The recombinant plasmid for protein purification was synthesized and sequence-verified by Sangon Biotech (Shanghai) Co., Ltd. (Shanghai, China). The target gene was cloned into the pET-28a(+) expression vector, which carries a C-terminal 6 × His-tag for subsequent Ni-NTA affinity chromatography purification.

MmpL3 (UniProt ID: P9WJV5, residues 1–753) was expressed in *E. coli* C43 (DE3) at 25 °C, 0.4 mM IPTG. Cells were lysed in 100 mL PBS + 150 mM NaCl using a high-pressure homogenizer and sonication for 10 min at 4 °C. Membranes were pelleted (37,000 rpm, 1 h), solubilized in 50 mL PBS + 150 mM NaCl + 1% LMNG, and clarified (41,000 rpm, 1 h). The supernatant was loaded onto a 5 mL Ni-NTA column, washed with 30 mL PBS + 150 mM NaCl + 0.1% LMNG + 15 mM imidazole and 30 mL + 30 mM imidazole, and eluted with 12 mL PBS + 150 mM NaCl + 0.1% LMNG + 250 mM imidazole. SDS-PAGE showed >90% purity.

PPE57 (UniProt ID: Q50703, residues 1–176) and PPE57_S36A_, PPE57_R157A_, PPE57_E168A_, PPE57_G175A_ were expressed in *E. coli* B21 (DE3) at 25 °C, 0.25 mM IPTG. Cells were lysed in 100 mL PBS + 150 mM NaCl with a high-pressure homogenizer and 10 min ultrasound (4 °C) and clarified (15,000 rpm, 1 h). The supernatant was loaded onto a 5 mL Ni-NTA column, washed with 30 mL PBS + 150 mM NaCl + 10 mM imidazole and 30 mL + 50 mM imidazole, and eluted with 12 mL PBS + 150 mM NaCl + 250 mM imidazole. SDS-PAGE showed >90% purity.

MmpL3-GST-D1 (UniProt ID: P9WJV5, residues 32–187) and MmpL3-GST-D2 (UniProt ID: P9WJV5, residues 419–560) were expressed in *E. coli* B21 (DE3) at 25 °C, 0.25 mM IPTG. Cells were disrupted by ultrasound, and the lysate was clarified by centrifugation at 15,000 rpm for 30 min at 4 °C. The supernatant was loaded onto a Glutathione Sepharose 4B column equilibrated with PBS. After washing with 10 column volumes of PBS, bound proteins were eluted with elution buffer (10 mM reduced glutathione, 50 mM Tris-HCl pH 8.0, 150 mM NaCl). SDS-PAGE showed >90% purity.

### 2.13. ELISA to Detect the Affinity of MmpL3 and TMM

Biotinylated TMM (Cat#890809P, Sigma, St. Louis, MO, USA) was prepared by labeling TMM with EZ-Link Hydrazide-Biotin (Cat#21339, Thermo Fisher, Waltham, MA, USA). Then, 1 µg/mL of biotinylated TMM was coated onto a streptavidin-treated ELISA plate (Cat#SEKF117, Solarbio, Beijing, China) and incubated overnight at 4 °C. After washing with PBS, the plate was blocked with 5% bovine serum albumin in PBS for 1 h at 37 °C. After washing with PBS, 6 × His-MmpL3 protein was added at a 2-fold dilution in the presence or absence 2 µg/mL PPE57 protein or OVA control. HRP-conjugated anti-His antibody (1:8000; Cat#HRP-66005, Proteintech, Wuhan, China) was used as the secondary antibody. 3,3′,5,5′-Tetramethylbenzidine (Cat#PR1200, Solarbio, China) was added to each well as the substrate for 10 min, and 2 M H_2_SO_4_ was added to stop the reaction. The optical density was measured at 450 nm and 630 nm.

### 2.14. Detection of Lipid Droplet Changes in Cells

After 48 h of PMA induction, THP-1 cells were infected at an MOI of 5. After 48 h of infection, the cell culture medium was removed, and the cells were washed with ice-cold PBS. The cells were then fixed with paraformaldehyde for 10 min. Subsequently, the cells were permeabilized with 0.1% Triton X-100 (Cat#A417820, Sangon, China) and washed three times with PBS. The cells were blocked with 1% BSA for 1 h. Then, 80 µL of the primary antibody anti-Mycobacterium tuberculosis (Cat#ab905, Abcam, Cambridge, UK) was added, and the cells were incubated overnight at 4 °C. After washing twice with PBST, the secondary antibody (diluted in blocking solution at a ratio of 1:500) was added, and the cells were incubated in the dark at room temperature for 30 min. The cells were washed three times with PBST. The BODIPY 493/503 (Cat#C2053S, Beyotime, Shanghai, China) fluorescent probe was added for lipid droplet staining. During this process, DAPI solution (Cat#C1002, Beyotime, China) was added for nuclear staining. After incubation in the dark at room temperature, the cells were mounted and imaged using a confocal fluorescence microscope (Olympus FV3000, Tokyo, Japan).

### 2.15. Isolation of RNA and RT-qPCR

Total RNA was extracted using Trizol (Cat#15596026, Invitrogen, Waltham, MA, USA). Using the HiScript III 1st Strand cDNA Synthesis Kit (Cat#R312-01, Vazyme, Nanjing, China), 1 µg of isolated RNA was immediately reverse-transcribed into cDNA. For RT-qPCR analysis, the Taq Pro Universal SYBR qPCR Master Mix (Cat#Q712-02, Vazyme, China) was used. Data from three independent experiments were used for statistical analysis.

### 2.16. Western Blotting

Whole-cell lysates were obtained using Western lysate (Cat#P0013, Beyotime, China). Then, 15 μg of total protein was separated by SDS-PAGE (10% acrylamide gels) and transferred onto a PVDF membrane (Cat# IPVH00010, Millipore, Burlington, MA, USA). After blocking with 5% skimmed milk for 2 h, the membrane was incubated with antibodies against the PPAR-γ (Cat#AF7797, Beyotime, China), MmpL3 (this study), and PPE57 (this study) antibodies. After washing, membranes were incubated with HRP-conjugated goat anti-rabbit IgG antibodies (Cat#SA00001-2, Proteintech, China) or HRP-conjugated goat anti-mouse IgG antibodies (Cat#SA00001-1, Proteintech, China) diluted 1:8000 in TBST (Tris-buffered saline, pH8.0, 0.05% Tween 20) containing 5% skimmed milk. After washing, the bands were developed using an ultrasensitive ECL substrate (Cat#SQ201, Epizyme, Shanghai, China).

### 2.17. Statistical Analysis

All statistical analyses were performed using GraphPad Prism 9 (GraphPad Software Inc., La Jolla, CA, USA). Normality and homogeneity of variances were assessed using Shapiro–Wilk and Bartlett’s tests, respectively. Data that passed these assumptions were analyzed using parametric tests (*t*-test or one-way ANOVA with Tukey’s honest significant difference (HSD) post hoc test for multi-group pairwise comparisons); non-parametric alternatives (Mann–Whitney U or Kruskal–Wallis with Dunn’s post hoc test) were used when assumptions were not met. *p* < 0.05 was considered to indicate statistical significance.

## 3. Results

### 3.1. Impact of PPE57 on M. tuberculosis H37Rv Colony Morphology and Biofilm Development

**Figure 1:**
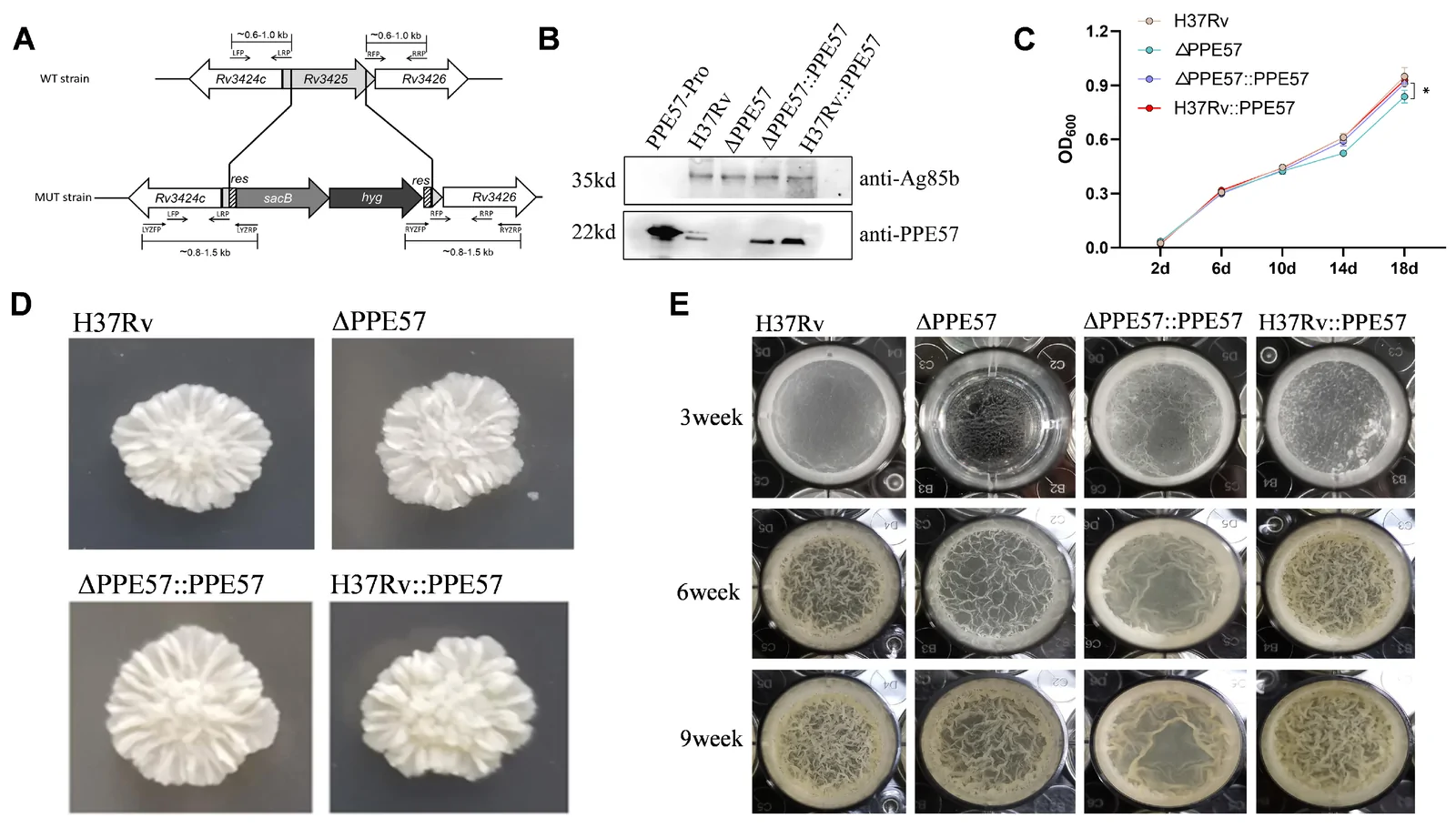
PPE57 affects the growth state of bacteria. (**A**) Schematic diagram of knockout strain construction via specialized phage transduction. (**B**) WB validation of PPE57 expression in Δ*PPE57*, Δ*PPE57::PPE57*, and *H37Rv::PPE57* strains. (**C**) Growth curve in 7H9-OADC liquid medium. (**D**) Colony morphology on 7H10-ODAC solid medium at 37 °C for 6 weeks. (**E**) Biofilm formation in Sauton’s medium at 37 °C after 3, 6, and 9 weeks. Data are shown as mean ± SD, *n* = 3. Statistical analysis was performed using two-way ANOVA (*, *p* < 0.05).

To investigate the role of PPE57 in *M. tb* H37Rv, we generated a knockout strain using specialized phage transduction (Figure 1A). For complementation and overexpression, the *pMV361-ppe57* plasmid was introduced into the Δ*PPE57* and wild-type strains, respectively. The knockout (Δ*PPE57*), complementation (Δ*PPE57::PPE57*), and overexpression (*H37Rv::PPE57*) strains were confirmed by Western blotting (WB) (Figure 1B) and RT-qPCR (Figure S1).

We next examined the bacterial growth in standard 7H9-OADC medium supplemented with kanamycin. In standard 7H9-OADC liquid medium, *Δ*PPE57** exhibited a slight but reproducible growth delay starting from approximately day 12, and by day 18, its OD600 remained significantly lower than that of the wild-type strain. In contrast, *H37Rv* and *H37Rv::PPE57* grew at comparable rates throughout the observation period (Figure 1C). Colony morphology was also assessed on solid medium. All strains displayed the typical irregular, wrinkled, acne-like appearance. However, compared with the wild-type, *Δ*PPE57** formed colonies with fewer and less pronounced wrinkles. Complementation of PPE57 restored this morphological phenotype. Conversely, the overexpression strain produced colonies with more pronounced wrinkles and a thicker center relative to the wild-type (Figure 1D). Regarding the function of biofilm formation, after 3 weeks of growth, Δ*PPE57* grew predominantly as a planktonic form without forming a distinct biofilm, while the other strains formed a thin pellicle. At 6 and 9 weeks, Δ*PPE57* began to show signs of biofilm formation and trended toward wild-type-like pellicle development, although it remained less robust than the complemented strain. At all-time points examined, *H37Rv::PPE57* developed a thicker and more extensive biofilm meshwork than the wild-type (Figure 1E). Together, these results demonstrate that PPE57 is essential for normal growth, proper colony morphology, and biofilm formation in *M. tb*. Since these phenotypes are closely linked to cell envelope integrity, we next investigated whether PPE57 affects cell wall thickness.

### 3.2. PPE57 Deletion Leads to Changes in M. tb Cell Wall Thickness and Permeability

**Figure 2:**
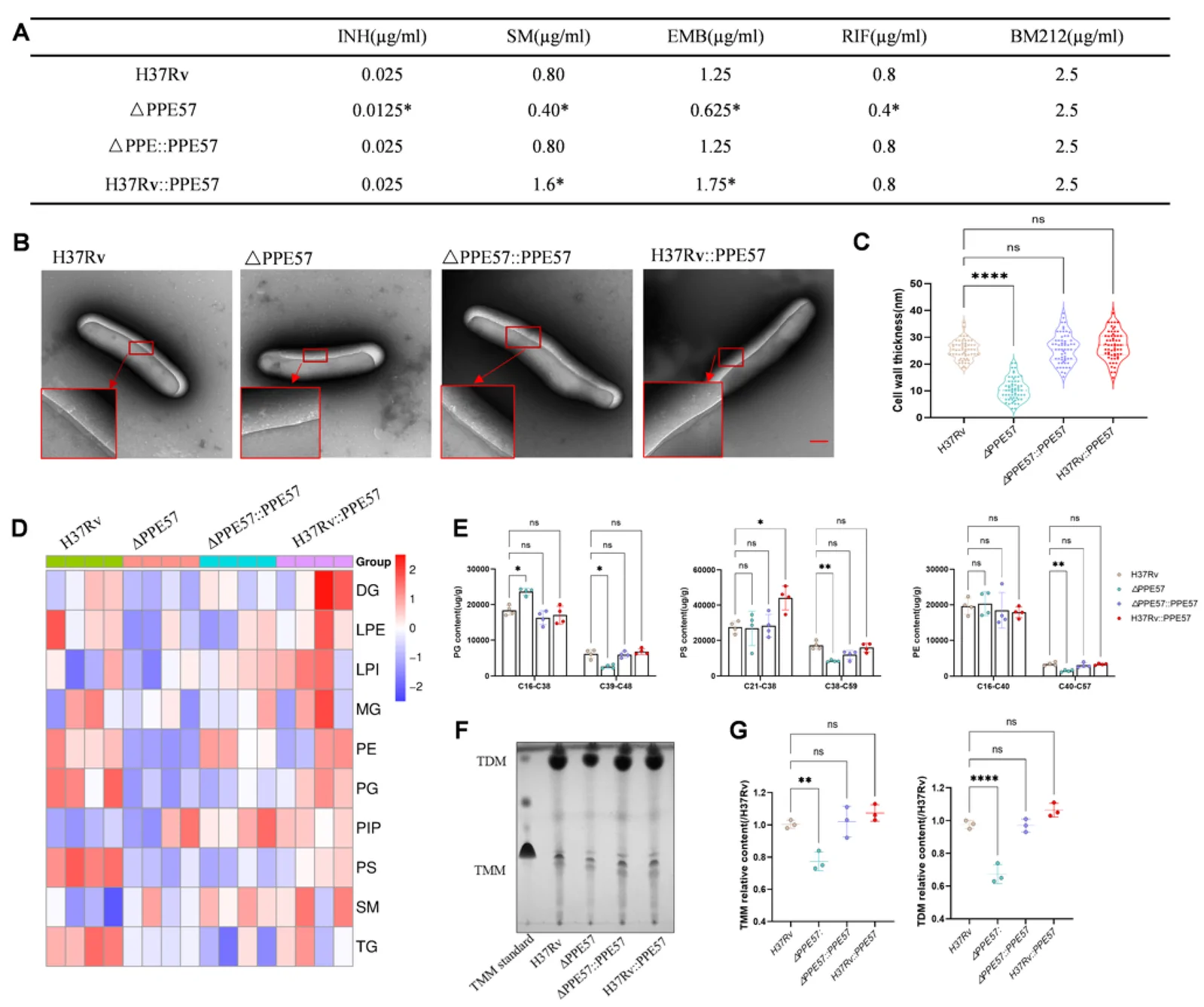
PPE57 deficiency remodels the cell wall and metabolomic landscape of *M. tb* (**A**) Minimum inhibitory concentration (MIC) values of the indicated strains. (**B**) Representative transmission electron microscopy images of bacterial cell wall thickness (Scale bar, 500 nm). (**C**) Statistical analysis of cell wall thickness of different strains Data are shown as mean ± SD from three independent biological experiments (*n* = 3). For each experiment, 100 randomly selected cells were quantified per condition. (**D**) Heat map of lipid content in different subtypes of bacterial strains. (**E**) Content of lipids with different chain lengths in PG, PS, and PE. (**F**) Thin-layer chromatography (TLC) analysis of TDM and TMM content in different strains. (**G**) Statistical analysis of TDM and TMM content. Data are shown as mean ± SD, *n* = 4/3. Statistical analysis was performed using one-way ANOVA (ns, No significance; *, *p* < 0.05, **, *p* < 0.01, ****, *p* < 0.0001).

To determine whether the phenotypic changes described above reflect alterations in cell envelope structure, we next examined cell wall thickness and permeability. We first performed minimum inhibitory concentration (MIC) tests for anti-tuberculosis drugs using the *H37Rv*, Δ*PPE57*, Δ*PPE57::PPE57*, and *H37Rv::PPE57* strains. The results revealed that the deletion of PPE57 led to increased susceptibility to these drugs (Figure 2A). Given that these drugs act through distinct mechanisms, we hypothesize that the absence of PPE57 may alter the permeability or thickness of the bacterial cell wall, thereby facilitating drug entry and enhancing bactericidal activity. To verify this hypothesis, we examined the four strains in logarithmic phase by transmission electron microscopy (TEM). In negatively stained samples, the cell wall contours of the bacteria were clearly visible. Compared with the wild-type and complemented strains, Δ*PPE57* exhibited significantly reduced cell wall thickness. The overexpressing strain *H37Rv::PPE57* did not show increased cell wall thickness relative to the wild-type, consistent with the lack of enhanced drug resistance observed in the MIC assays (Figure 2B,C).

To further assess whether PPE57 influences cell wall permeability, we performed ethidium bromide (EB) uptake and efflux assays using the BCG strain, which naturally lacks PPE57, along with BCG harboring an empty vector (BCG-Vec) and BCG overexpressing PPE57 (BCG-PPE57). In the EB uptake assay, *BCG-PPE57* exhibited significantly weaker intracellular fluorescence intensity than the control strains, indicating reduced EB accumulation, which is consistent with decreased cell wall permeability. Conversely, in the efflux assay, *BCG-PPE57* showed slower fluorescence decay, suggesting impaired EB efflux (Figure S2A,B). These results together suggest that PPE57 reduces the overall cell wall permeability, likely by promoting a denser cell envelope architecture.

Collectively, the observed changes in drug susceptibility, cell wall thickness, and permeability suggest that PPE57 may influence the lipid composition or content of the mycobacterial cell wall, leading to the phenotypic differences described above.

### 3.3. The Absence of PPE57 Reduces the Lipid Content and Lipid Metabolism Levels in M. tb

To comprehensively assess the impact of PPE57 on bacterial lipid content and composition, we performed high-resolution, broad-spectrum lipidomics with absolute quantification on bacteria harvested at logarithmic phase, followed by detailed comparisons of bacterial lipid content, types, and chain lengths. Deletion of PPE57 profoundly affected the lipid profile of *M. tb*, leading to a marked reduction in total lipid content. In contrast, the complemented and overexpression strains exhibited lipid levels comparable to those of the wild-type strain (Figure 2D). Compared with the wild-type, the Δ*PPE57* strain showed significantly decreased levels of total lipids and major lipid components, including diacylglycerol (DG), triglyceride (TG), and phosphatidylglycerol (PG) (Figure 2D). In addition to lipid abundance, acyl chain length is another critical determinant of lipid function, as it influences membrane thickness, permeability, and the activity of lipid transporters and associated proteins. Phospholipids, such as phosphatidylglycerol (PG), phosphatidylserine (PS), and phosphatidylethanolamine (PE), are major components of the mycobacterial cell wall, and alterations in their chain length may affect bacterial permeability. For PG, PS, and PE, the Δ*PPE57* strain exhibited significantly reduced levels of relatively long-chain species compared with the wild-type strain, whereas no differences were observed in the complemented or overexpression strains. Conversely, the Δ*PPE57* strain showed a significant increase in short-chain PG species compared with the wild-type, suggesting a shift toward shorter acyl chains that may contribute to altered membrane fluidity and permeability (Figure 2E). Therefore, these findings suggest that PPE57 deficiency impairs lipid utilization or transport in mycobacteria, leading to a shortage of lipid building blocks for cell wall assembly and contributing to the observed changes in cell wall permeability and thickness.

To further explore the molecular basis of the observed lipid alterations, we performed RNA sequencing analysis. KEGG pathway analysis revealed a significant reduction in pathways related to secondary metabolism, ABC transporters, and fatty acid degradation in the Δ*PPE57* strain (Figure S3A). Given the well-established role of ABC transporters in mycobacterial lipid export, we next examined the expression of MmpL3, which encodes a key transporter responsible for shuttling lipids from the inner membrane to the outer membrane. Notably, MmpL3 expression was significantly upregulated in the knockout strain (Figure S3B,C).

MmpL3 is essential for the outer membrane lipid synthesis in mycobacteria. To assess its functional relevance, we performed thin-layer chromatography to analyze the trehalose monomycolate (TMM) and trehalose dimycolate (TDM) levels. Compared with the wild-type and complemented strains, the Δ*PPE57* strain exhibited markedly reduced levels of both TMM and TDM (Figure 2F,G). Collectively, these findings suggest that PPE57 modulates mycolic acid transport, likely through its functional association with MmpL3.

### 3.4. PPE57 Works in Concert with the Lipid Transport Protein MmpL3

**Figure 3:**
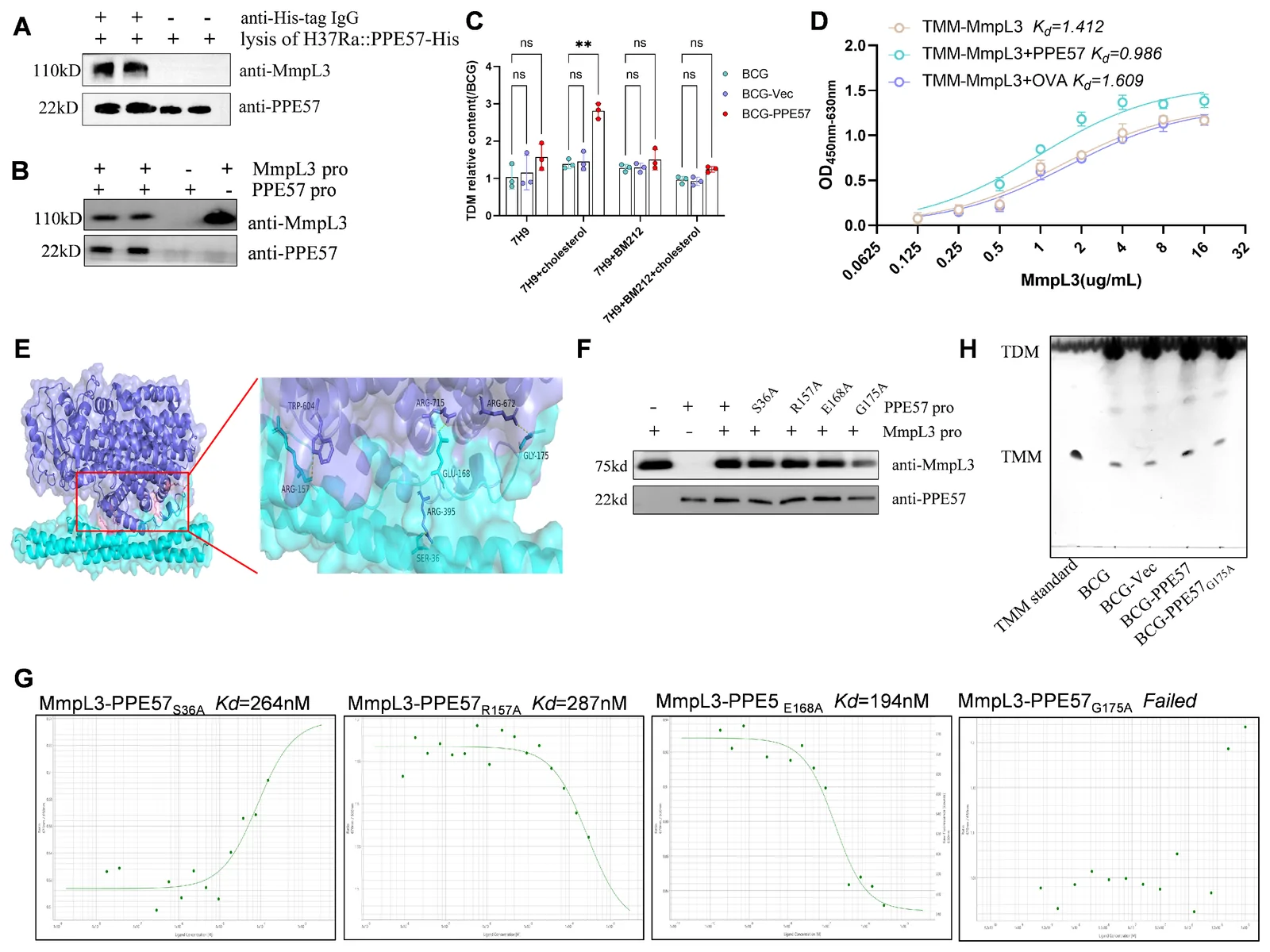
PPE57 works in concert with MmpL3. (**A**) Co-IP validation of the interaction between PPE57 and MmpL3. (**B**) In vitro immunoprecipitation confirming the direct binding of PPE57 and MmpL3. (**C**) Changes in the content of TDM in BCG strains treated with the MmpL3 inhibitor BM212. (**D**) Binding ability of MmpL3 to TMM in the presence or absence of PPE57. Data were fitted to a one-site specific binding hyperbola (nonlinear regression). (**E**) Molecular docking of PPE57 (cyan) and MmpL3 (blue-purple), with the interface shown as pink sticks. (**F**) Immunoprecipitation of MmpL3 with wild-type or point-mutated PPE57 proteins. (**G**) Binding constants (Kd) of different PPE57 point mutants with MmpL3 determined by MST. (**H**) TLC analysis of TMM/TDM levels in BCG strains expressing the G175A mutant of PPE57. Data are shown as mean ± SD, *n* = 3. Statistical analysis was performed using one-way ANOVA (ns, No significance; **, *p* < 0.01).

To further investigate the functional association between PPE57 and MmpL3, we first investigated the physical interaction between PPE57 and MmpL3. PPE57 was overexpressed in the *M. tuberculosis* H37Ra strain, and co-immunoprecipitation (Co-IP) assays were performed to identify PPE57-interacting proteins. The results showed that MmpL3 specifically interacts with PPE57 (Figure 3A). To further validate this interaction, we purified the PPE57 and MmpL3 protein (Figure S4A–C) and performed in vitro immunoprecipitation (Figure 3B), as well as GST pull-down assays with its extracellular domains (D1 and D2). The D1 and D2 extracellular/periplasmic domains of MmpL3 were selected for GST pull-down assays based on structural predictions indicating that these regions are exposed on the outer surface of the inner membrane and are therefore most likely to mediate interactions with accessory proteins such as PPE57 (Figure S4D), both of which confirmed the direct interaction between PPE57 and MmpL3. Moreover, microscale thermophoresis (MST) experiments demonstrated specific binding between MmpL3 and PPE57, with an estimated dissociation constant (Kd) of 18.6 nM (Figure S5A,B).

To gain molecular-level insights, we performed computational simulations of the PPE57–MmpL3 interaction. Multiple residues capable of forming hydrogen bonds were identified at the interface, and the predicted binding energy and dissociation constant were −9.9 kcal/mol and 5.5 × 10^−8^ M, respectively, values that are in close agreement with the MST data. Molecular dynamics simulations over 500 ns revealed stable complex formation, as assessed by the root-mean-square deviation (RMSD), radius of gyration (RG), and molecular surface area (MSA) analyses (Figure S5C–E). The periodic fluctuations observed during the simulation further supported the stability of the interaction.

To determine whether PPE57 functions in concert with MmpL3, we treated bacterial cultures with BM212, a specific MmpL3 inhibitor. In contrast to untreated conditions, overexpression of PPE57 did not lead to increased TDM levels in the presence of BM212 (Figure 3C), indicating that PPE57 cannot drive lipid transport independently but rather enhances cell wall lipid content by cooperating with MmpL3-mediated transport. Furthermore, given that MmpL3 binds TMM for transport to the cell wall, we examined whether PPE57 influences this interaction. Notably, the addition of PPE57 significantly enhanced the binding affinity of MmpL3 for TMM (Figure 3D). Collectively, these results support a model in which PPE57 promotes mycolic acid transport by physically associating with MmpL3 and enhancing its substrate-binding capacity.

Based on the binding interface between PPE57 and MmpL3 predicted by HDOCK (Figure 3E), we constructed four point-mutated PPE57 variants (PPE57_S36A_, PPE57_R157A_, PPE57_E168A_, and PPE57_G175A_), which were purified as recombinant proteins (Figure S4E). Then, we measured their binding affinity for MmpL3 using an immunoprecipitation assay, and the results showed that the binding ability of PPE57_G175A_ to MmpL3 was significantly reduced (Figure 3F).

We further measured their binding affinities for MmpL3 using MST. The resulting dissociation constants (Kd) were as follows: PPE57_S36A_, 264 nM; PPE57_R157A_, 287 nM; PPE57_E168A_, 194 nM; whereas PPE57_G175A_ exhibited negligible binding, precluding curve fitting (Figure 3G). Compared with the Kd of wild-type PPE57 (18.6 nM), the three mutants PPE57_S36A_, PPE57_R157A_, and PPE57_E168A_ all showed substantially reduced binding affinities (Figure 3G). Notably, the G175A mutation completely abolished the interaction, suggesting that the predicted hydrogen bond involving G175 represents a critical determinant for PPE57-MmpL3 binding.

To further validate the functional significance of the G175 residue identified above, we examined the impact of the G175A mutation on mycolic acid transport. Using thin-layer chromatography (TLC), we analyzed the TMM and TDM levels in strains expressing the PPE57_G175A_ mutant. Compared with wild-type PPE57, the G175A mutation led to a marked reduction in both TMM and TDM content, consistent with the impaired binding affinity observed in the MST assays (Figure 3H). These results further support that the G175-mediated interaction between PPE57 and MmpL3 is critical for efficient mycolic acid transport and outer membrane lipid assembly.

### 3.5. PPE57 Promotes Cholesterol Utilization in M. tb

Previous studies have established a functional link between cholesterol utilization and mycobacterial cell wall lipid metabolism: cholesterol catabolism provides precursors for the synthesis of virulence lipids such as PDIM, while the cholesterol import system Mce4 functionally interacts with the lipid transport machinery [21,29]. These findings suggest that cholesterol utilization and cell wall lipid metabolism are mechanistically coupled in mycobacteria. Given our observation that PPE57 modulates mycolic acid transport via MmpL3, we next investigated whether this regulatory function extends to cholesterol acquisition.

**Figure 4:**
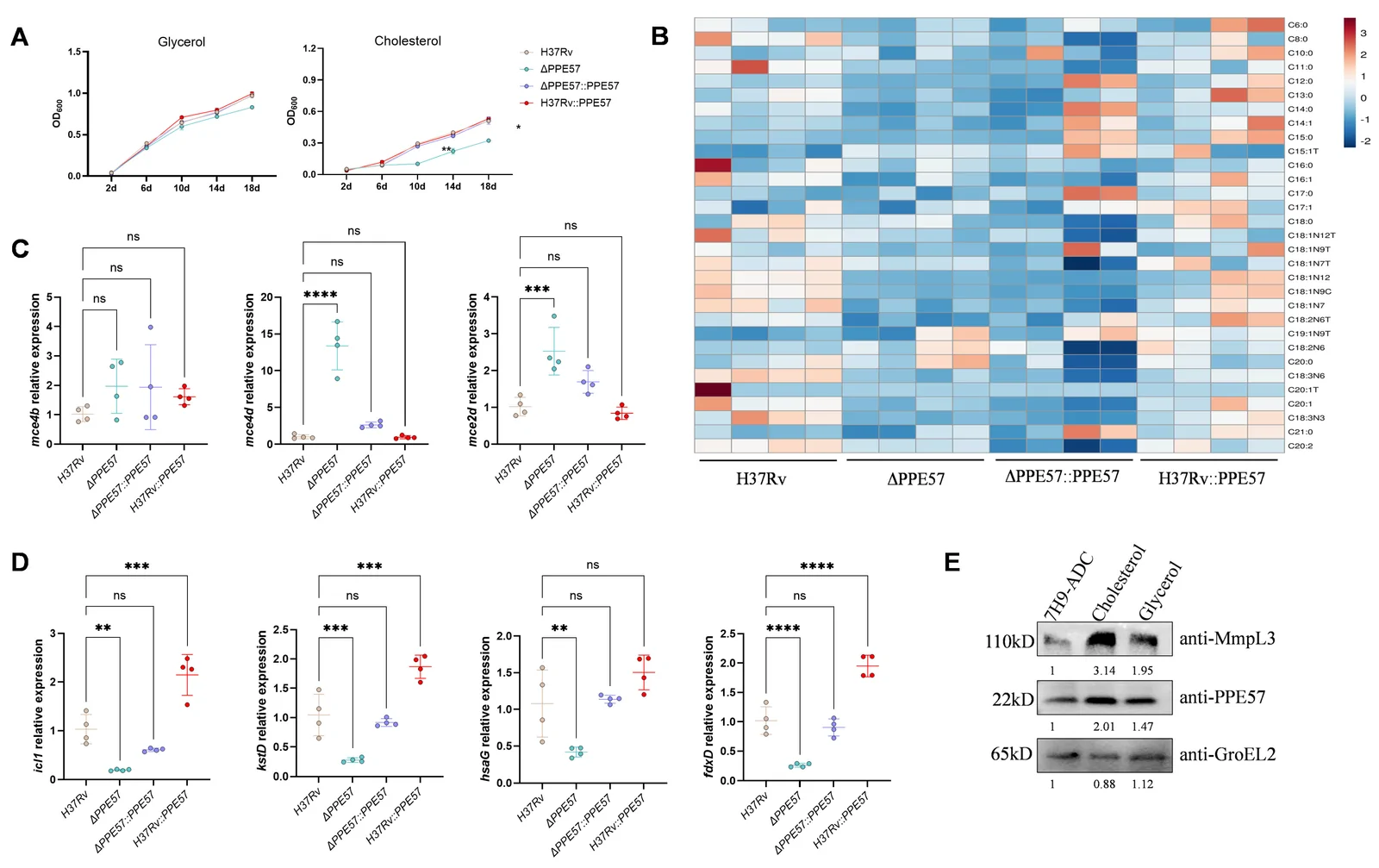
PPE57 affected the cholesterol uptake ability of *M. tb*. (**A**) Growth curves of the indicated strains in Minimal Nutrient Medium (MM) with a single carbon source. (**B**) Metabolic flux detection after culture with ^13^C-labeled cholesterol. (**C**,**D**) Expression changes of genes involved in cholesterol uptake and degradation. (**E**) WB analysis of PPE57 and MmpL3 expression after culture with the sole carbon source. Data are shown as mean ± SD, *n* = 4. Statistical analysis was performed using one-way ANOVA (ns, No significance; *, *p* < 0.05, **, *p* < 0.01, ***, *p* < 0.001, ****, *p* < 0.0001).

To assess whether PPE57 influences cholesterol utilization, we first compared the growth of different strains in minimal medium containing cholesterol as the sole carbon source. Under this condition, the Δ*PPE57* strain exhibited significantly restricted growth compared with the wild-type and complemented strains, whereas in complete medium or in medium containing glycerol as the sole carbon source, Δ*PPE57* showed a slight but reproducible growth defect starting from day 10, although this difference was not statistically significant. In complete medium, no growth difference was observed among the strains (Figure 4A). To directly evaluate cholesterol uptake capacity, we overexpressed PPE57 in the BCG strain (which naturally lacks PPE57) and used NBD-cholesterol as a fluorescent probe. *BCG-PPE57* cells accumulated significantly more cholesterol over time, accompanied by increased cholesterol consumption from the culture supernatant (Figure S6). Consistent with these findings, when bacteria were cultured with ^13^C-labeled cholesterol as the sole carbon source, the Δ*PPE57* strain incorporated substantially less labeled lipid into its cellular components (Figure 4B). Collectively, these results indicate that PPE57 enhances the ability of mycobacteria to acquire and utilize cholesterol.

We next investigated the molecular basis for this functional difference. Given that cholesterol uptake in mycobacteria is primarily mediated by Mce transporters, using RT-qPCR, we examined the expression of *mce4b*, *mce4d*, and *mce2d*—exactly the genes related to the KEGG change pathway and cholesterol uptake. While *mce4b* expression remained unchanged, *mce4d* and *mce2d* were significantly upregulated in the Δ*PPE57* strain (Figure 4C), likely representing a compensatory response to impaired cholesterol uptake. Consistent with reduced cholesterol availability, the expression of genes involved in intracellular cholesterol degradation—including *icl1*, a key regulator of the glyoxylate shunt, as well as *ktsD*, *hsaG*, and *fdxD*—were all significantly decreased in the Δ*PPE57* strain. Conversely, in the *H37Rv::PPE57* overexpression strain, the expression of icl1, ktsD, hsaG, and fdxD was significantly upregulated compared with the wild-type, further supporting a positive role for PPE57 in cholesterol catabolism (Figure 4D). Notably, when H37Rv was cultured with cholesterol as the sole carbon source, the expression levels of both PPE57 and MmpL3 were upregulated (Figure 4E), suggesting a potential link between the PPE57-MmpL3 transport axis and cholesterol utilization. These findings suggest that PPE57 not only facilitates cholesterol uptake but also influences downstream metabolic pathways, likely through its role in maintaining proper cell wall lipid composition via the MmpL3-dependent transport axis.

### 3.6. PPE57 Enhances Intracellular Survival by Promoting Host Lipid Droplet Formation via the PPAR-γ Pathway

**Figure 5:**
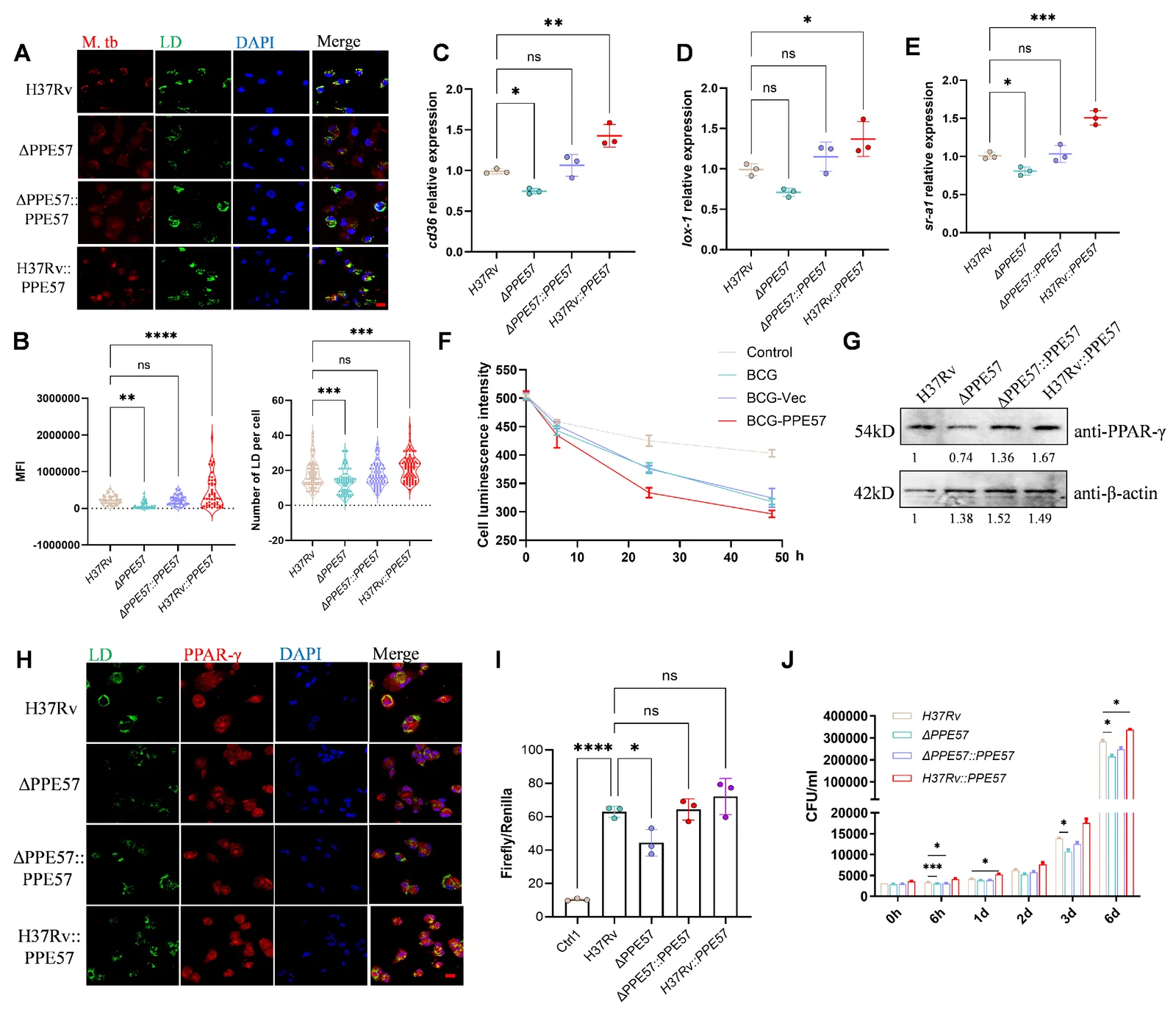
PPE57 affects the ability of bacteria to persist in host cells. (**A**) Lipid droplet formation in THP-1 cells 2 days post-infection with the indicated strains (Scale bar, 10 µm). (**B**) Statistical analysis of the average fluorescence intensity of lipid droplets and the average number of lipid droplets formed per cell. Data are shown as mean ± SD from three independent biological experiments (n = 3). For each experiment, 100 randomly selected cells were quantified per condition. (**C**–**E**) Expression levels of cholesterol uptake genes in THP-1 cells 48 h post-infection with the indicated strains. (**F**) NBD-cholesterol uptake in THP-1 cells after infection with BCG-related strains. (**G**) WB of PPAR-γ in THP-1 cells 48 h after infection. (**H**) Immunofluorescence detection of lipid droplet formation and PPAR-γ in THP-1 cells 2 days post-infection (Scale bar, 10 µm). (**I**) Activation of PPAR-γ gene transcription in cells at 24 h post-infection. (**J**) Bacterial counts of the indicated strains in infected THP-1 cells. Data are shown as mean ± SD, *n* = 3. Statistical analysis was performed using one-way ANOVA (ns, No significance; *, *p* < 0.05, **, *p* < 0.01, ***, *p* < 0.001, ****, *p* < 0.0001).

Having established that PPE57 enhances bacterial cholesterol acquisition, we next asked whether this bacterial trait influences host–pathogen interactions, particularly the metabolic reprogramming of macrophages. We therefore investigated whether PPE57 affects macrophage cholesterol uptake and lipid droplet formation. We first investigated lipid droplet formation in macrophages following infection. Compared with the wild-type strain, the Δ*PPE57* strain induced significantly fewer lipid droplets, and the that droplets formed were smaller in size. In contrast, the PPE57-overexpressing strain induced more lipid droplets than the wild-type strain (Figure 5A,B). Lipid droplets are intracellular organelles composed primarily of neutral lipids, including triglycerides (TAG) and cholesteryl esters (TC). To further characterize the impact of PPE57 on lipid droplet accumulation, we measured the levels of TAG, TC, and free fatty acids (FFAs) in the infected cells. After 48 h of infection in THP-1 cells, the *BCG-PPE57* strain induced significantly higher levels of TAG and TC compared with the vector control, a finding that was also observed in the RAW264.7 cells (Figure S7A,B). FFA levels showed a significant increase at 24 h post-infection; however, no significant difference was observed between *BCG-PPE57* and control strains at later time points (Figure S7A,B).

To elucidate the mechanism by which PPE57 promotes lipid droplet accumulation in macrophages, we examined whether this effect results from altered cholesterol uptake or efflux in host cells. We evaluated the expression of genes involved in cholesterol uptake and efflux in THP-1 cells infected with different bacterial strains for 48 h. Compared with the wild-type and complemented strains, macrophages infected with the Δ*PPE57* strain exhibited significantly reduced expression of the uptake-related genes *sr-a1*, *cd36* and *lox-1* and showed similar downward trends (Figure 5C–E). Conversely, infection with PPE57-overexpressing strains led to significant upregulation of *cd36*, *sr-a1*, and lox-1 expression (Figure 5C–E). In contrast, the expression of cholesterol efflux-related genes *abca1* and *abcg1* showed no consistent pattern of change (Figure S7C), suggesting that the increased intracellular lipid content in infected macrophages primarily results from enhanced uptake rather than impaired efflux. To directly assess cholesterol uptake capacity, as we were unable to monitor the non-inactivated culture supernatant from infections with *M. tb*, we instead measured the residual NBD-cholesterol remaining in the supernatant following infection with BCG, which naturally lacks PPE57. Macrophages infected with PPE57-overexpressing strains exhibited significantly enhanced cholesterol uptake, further supporting this conclusion (Figure 5F).

Given that PPAR-γ is a master regulator of cholesterol uptake genes, including *cd36*, we next examined its involvement. Previous studies have reported that mycobacteria modulate host lipid metabolism via PPAR-γ. Consistent with our hypothesis, PPAR-γ expression was significantly decreased in macrophages infected with the Δ*PPE57* strain and increased upon infection with PPE57-overexpressing strains (Figure 5G,H). Moreover, a dual-fluorescence reporter assay revealed that PPAR-γ transcriptional activity was significantly reduced in cells infected with the knockout strain (Figure 5I).

Collectively, these results indicate that, in addition to enhancing bacterial cholesterol acquisition, PPE57 also promotes macrophage cholesterol uptake by activating the PPAR-γ signaling pathway, thereby leading to increased intracellular lipid content and lipid droplet formation.

Given that lipid droplets serve as a nutrient reservoir for intracellular *M. tb*, we next examined whether PPE57-mediated enhancement of macrophage cholesterol uptake and lipid droplet formation translates into improved bacterial survival within host cells. To directly test this possibility, we examined the intracellular survival of different M. tb strains in THP-1 macrophages. Following infection with the *H37Rv*, Δ*PPE57*, Δ*PPE57::PPE57*, and *H37Rv::PPE57* strains, the bacterial burden of the Δ*PPE57* strain was significantly reduced at 3 and 6 days post-infection compared with the wild-type and complemented strains (Figure 5J). Similar results were obtained using BCG strains in both THP-1 and RAW264.7 macrophages. In the absence of PPE57, mycobacterial retention within the macrophages was markedly decreased, whereas overexpression of PPE57 enhanced bacterial persistence in these cells (Figure S7D). Collectively, our results indicate that PPE57 activates the PPAR-γ signaling pathway in macrophages, leading to enhanced cholesterol uptake, accumulation of TC and TAG, and increased lipid droplet formation.

### 3.7. PPE57 Promotes Bacterial Infection in Mice

**Figure 6:**
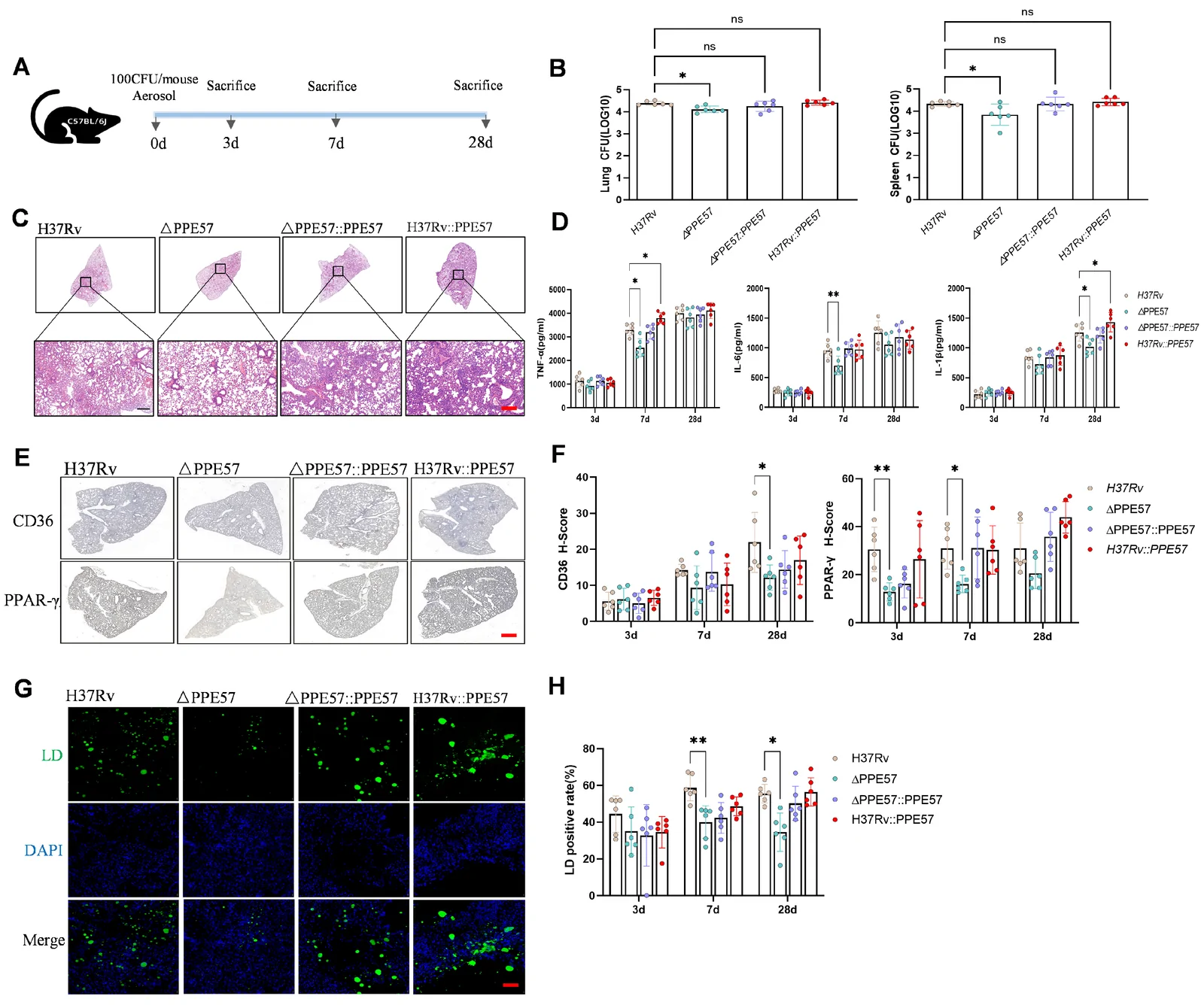
PPE57 promotes bacterial infection and lipid uptake in mice. (**A**) Schematic diagram of the mouse infection experiment schedule. (**B**) Bacterial load in the lungs and spleen after bacterial infection. (**C**) H&E staining of lung sections at 28 dpi (Scale bar, 50 µm). (**D**) Secretion levels of TNF-α, IL-6, and IL-1β in the lungs. (**E**) Representative images of the immunohistochemistry of lipid associated protein CD36 and PPAR-γ after infection (Scale bar, 500 µm). (**F**) Statistical analysis of H-Score for CD36 and PPAR-γ post-infection. (**G**) Immunofluorescence of lipid droplet-positive cells at 28 dpi (Scale bar, 50 µm). (**H**) Statistical analysis of lipid droplet-positive cells in mouse lungs. Data are shown as mean ± SD, n = 6. Statistical analysis was performed using one-way ANOVA (ns, No significance; *, *p* < 0.05, **, *p* < 0.01).

To elucidate the functional role of PPE57 during *M. tb* infection in vivo, C57BL/6J mice were challenged via aerosol inhalation with approximately 100 colony-forming units (CFU) of *H37Rv*, Δ*PPE57*, Δ*PPE57::PPE57*, *H37Rv::PPE57*, and euthanized at 3, 7, and 28 days post-infection (dpi) (Figure 6A). At 28 dpi, the pulmonary bacterial loads in mice infected with the Δ*PPE57* mutant were significantly reduced compared to those infected with the wild-type or complemented strains, and in the spleen, a significant reduction was also seen at 28 dpi, indicating that PPE57 deficiency markedly impairs bacterial colonization and persistence in the lung. No significant increase was observed in the overexpression group (Figure 6B).

Lung pathology was assessed at 3, 7, and 28 dpi. No lesions were observed at 3 dpi. At 7 dpi, Δ*PPE57*-infected mice showed milder inflammation and better-preserved alveolar architecture, whereas PPE57-overexpressing mice exhibited more severe inflammation and caseous necrosis. By 28 dpi, all groups had pathological changes and lymphocyte infiltration, but the degree of caseous necrosis in Δ*PPE57* infected mice was significantly milder than that in other groups (Figure 6C).

The secretion of pro-inflammatory cytokines by lung cells was examined. At 7 dpi, the secretion of TNF-α and IL-6 in the Δ*PPE57* group was significantly lower than in the wild-type group. At 28 dpi, the secretion of IL-1β in the Δ*PPE57* group was significantly lower than that in the wild-type strain, while the overexpressing group showed significantly higher levels (Figure 6D).

Immunohistochemical examination of lipid droplet biogenesis-associated proteins demonstrated that CD36, a critical mediator of cholesterol uptake, was substantially downregulated in the Δ*PPE57* group at 28 dpi. Furthermore, although the PPAR-γ signaling pathway was markedly activated at 3 and 7 dpi, PPAR-γ expression levels remained suppressed in the Δ*PPE57* group compared to the wild-type counterpart (Figure 6E,F). Quantitative assessment of pulmonary lipid content revealed that the proportion of lipid-positive adipocytes was significantly reduced in the Δ*PPE57* group relative to the wild-type group at both 7 and 28 dpi; conversely, PPE57 overexpression failed to significantly elevate lipid accumulation (Figure 6G,H). Collectively, these findings demonstrate that PPE57 facilitates host lipid acquisition and bacterial proliferation in vivo by modulating cholesterol uptake machinery and lipid droplet formation pathways, thereby playing a pivotal role in the pathogenesis of *M. tb* infection.

## 4. Discussion

In this study, we identified the PE/PPE family protein PPE57 as a critical virulence determinant that promotes *M. tb* persistence through a coordinated mechanism. Central to this process is the physical interaction between PPE57 and MmpL3, which facilitates mycolic acid transport and enhances bacterial cholesterol acquisition. This bacterial trait, in turn, activates the host PPAR-γ pathway, leading to macrophage cholesterol uptake, lipid droplet accumulation, and inflammation. The resulting lipid droplets provide a nutrient reservoir for the bacteria, while the inflammatory environment supports bacterial persistence. Together, these findings establish PPE57 as a key molecular link connecting mycobacterial lipid metabolism with host nutrient exploitation and immune modulation.

MmpL3 is an essential inner membrane transporter responsible for TMM flipping from the inner leaflet to the outer leaflet of the inner membrane or delivering it to the periplasm [14,30]. This transport activity is critical for mycobacterial viability, as MmpL3 is required for growth in vitro, inside macrophages, and in infected mice. However, despite its established function as the TMM flippase, the full transport machinery that couples mycolate synthesis to MmpL3 export remains incompletely defined. Specifically, a critical unanswered question is whether additional accessory proteins are required to coordinate TMM delivery from MmpL3 to downstream processing complexes such as the Ag85 enzymes. Recent studies have revealed that MmpL3 does not function in isolation. Auxiliary components of the MmpL3 complex have been identified—including TtfA (Rv0383c) [31], which is essential for TMM transport, and MSMEG_5308, which stabilizes the complex under stress. Here, we demonstrate that PPE57 acts as an additional auxiliary component. The physical interaction between PPE57 and MmpL3 was validated by co-immunoprecipitation, MST, and mutational analyses. Specifically, the G175 residue was identified as critical for this interaction, likely contributing to the binding interface through backbone hydrogen bonding, as its mutation abolished both binding and functional complementation. Collectively, these findings expand the current understanding of mycobacterial cell wall biogenesis by revealing that a PE/PPE protein can function as an auxiliary component of the MmpL3 transport machinery, thereby filling a key gap in the TMM transport pathway.

Our identification of PPE57 as an accessory component of the MmpL3 transport machinery raises the question of how it relates to previously described auxiliary proteins, such as TtfA (Rv0383c) and MSMEG_5308. TtfA has been shown to be essential for TMM transport and physically interacts with MmpL3, whereas MSMEG_5308 stabilizes the MmpL3 complex under stress conditions [32]. We speculate that PPE57 may function in a distinct but complementary capacity: rather than being strictly essential for basal TMM transport, PPE57 appears to enhance transport efficiency and couples this activity to cholesterol utilization. It is possible that PPE57, TtfA, and MSMEG_5308 occupy different binding sites on MmpL3 or act at different stages of the transport cycle, forming a dynamic regulatory network that adjusts mycolic acid export in response to environmental cues. Future structural studies of the PPE57–MmpL3 complex will be necessary to determine whether PPE57 competes with or synergizes with these other accessory proteins. A certain degree of scatter around the fitted MST curve is intrinsic to this technique, particularly for detergent-solubilized membrane proteins, and does not preclude a nanomolar-range interaction. nM-range MST affinities have been reported for other mycobacterial PE/PPE protein interactions [33], and a multi-laboratory benchmark study highlighted the inherent variability of MST-derived Kd values [34]. Thus, our measured affinities are consistent with the literature and should be considered as estimates. Furthermore, the in vitro MST measurement was performed with detergent-solubilized proteins, which may not fully capture the stabilizing effects of the native membrane environment, substrate lipids, or additional protein cofactors. It is therefore conceivable that the PPE57–MmpL3 interaction is further stabilized in vivo by factors absent from the reconstituted system, and the observed binding affinity should be considered a conservative estimate of the true interaction strength.

In addition to its well-established role in TMM translocation, MmpL3 has also been implicated in heme acquisition via its N-terminal D1 domain. The D1 domain of MmpL3 has been shown to directly bind heme, and together with the secreted protein Rv0203, MmpL3 is proposed to function as a putative heme transporter [35]. Given that MmpL3 handles two distinct substrates—TMM and heme—we speculate that PPE57 may enhance the efficiency or specificity of TMM translocation, potentially by stabilizing the outward-facing conformation of MmpL3 required for substrate release. Alternatively, PPE57 might spatially organize the MmpL3 transport complex at the cell envelope assembly sites, thereby coupling TMM delivery to the Ag85 mycolyltransferases [36]. In the absence of PPE57, MmpL3 might shift its activity toward heme transport, or its overall transport efficiency could be compromised. While direct testing of this hypothesis awaits future investigation, this model offers a plausible mechanism for how a single transporter coordinates multiple essential functions in *M. tb*.

Cholesterol is a central carbon source for *M. tb* during chronic infection, fueling bacterial persistence and modulating host immunity [37]. Its uptake is primarily mediated by the Mce4 transport system, whose activity is regulated at multiple levels: cholesterol enhances the ATPase activity of MceG, binds the K^+^ importer CeoBC, and is directly recognized by Mce4C [38], while the transcriptional repressor Rv3575c fine-tunes Mce4 operon expression [39]. Our study identifies PPE57 as a key positive regulator of bacterial cholesterol acquisition. We propose that PPE57-mediated enhancement of MmpL3 activity creates a heightened metabolic demand for mycolate precursors, thereby linking cell wall biosynthesis to central carbon metabolism. This metabolic coupling is further supported by our observation that cholesterol utilization upregulates both PPE57 and MmpL3 expression (Figure 4E), suggesting a feed-forward regulatory loop that coordinates nutrient availability with envelope biogenesis. To satisfy this demand, the bacterium upregulates its cholesterol acquisition machinery (e.g., the Mce4 system), as evidenced by increased uptake of exogenous cholesterol. This feed-forward loop—where cell wall synthesis drives nutrient scavenging, and nutrient availability in turn fuels envelope biogenesis—represents an elegant adaptation for intracellular persistence [40,41]. This metabolic demand-driven nutrient scavenging represents an active bacterial adaptation that links envelope biogenesis to host resource exploitation, enabling long-term persistence in macrophages [42].

Beyond its role in bacterial cholesterol acquisition, PPE57 also increases macrophage cholesterol uptake and lipid droplet formation dependent on PPAR-γ, a master regulator of lipid metabolism. Previous studies have demonstrated that M. tb infection significantly upregulates PPAR-γ expression in macrophages [43], leading to increased expression of the scavenger receptor CD36 and subsequent intracellular lipid accumulation [44,45]. Our identification of PPE57 as an upstream regulator of PPAR-γ activity provides new mechanistic insights into how *M. tb* manipulates host lipid metabolism to facilitate intracellular survival. Notably, this host-directed effect appears distinct from the cell-intrinsic function of PPE57 in mycolic acid transport, suggesting that PPE57 coordinates bacterial envelope integrity with host niche construction through dual, functionally linked mechanisms.

To further validate the role of PPE57 in PPAR-γ activation, we note that while previous studies implicated mycolic acids in PPAR-γ signaling, our genetic dissection specifically identifies PPE57 as the bacterial determinant linking envelope lipid content to host metabolic reprogramming. We acknowledge that the precise molecular mediator requires further validation—future experiments with purified lipids or macrophage-specific PPAR-γ knockouts will establish definitive causality. This represents active metabolic engineering rather than passive immune evasion: by inducing foamy macrophage differentiation, *M. tb* creates a nutrient-rich niche that supports persistence [46]. The markedly reduced bacterial burden and attenuated pathology in PPE57-deficient infections demonstrate that this metabolic manipulation is essential for virulence, not merely ancillary.

By activating PPAR-γ in macrophages, PPE57 simultaneously expands the host lipid reservoir available to the bacterium and promotes an inflammatory environment that supports bacterial persistence while contributing to tissue pathology. The PPE57-induced lipid accumulation in the lungs correlates with elevated IL-1β levels, consistent with the emerging concept of lipid droplets as signaling hubs that potentiate NLRP3 inflammasome activation [47,48]. However, whether IL-1β production directly benefits bacterial persistence or represents collateral host tissue damage remains to be determined. Intriguingly, the temporal dynamics of PPAR-γ activation (peaking at 3–7 dpi) preceding peak lipid droplet accumulation (28 dpi) suggest that PPE57 initiates host metabolic reprogramming early during infection to establish a nutrient-replete niche for subsequent bacterial expansion. In tuberculosis, mycobacterial components such as ManLAM drive foam cell formation via TLR2/Dectin-2 signaling [49]. Our findings extend this paradigm by identifying PPE57 as a bacterial factor that promotes both lipid accumulation and inflammation in vivo. Notably, preliminary data indicate that PPE57 also engages TLR2 on macrophages, suggesting that this receptor links bacterial recognition to downstream metabolic and inflammatory reprogramming [50]. Thus, PPE57 promotes a self-reinforcing loop in which PPAR-γ-driven lipid droplet accumulation and NLRP3-mediated IL-1β secretion together create a host environment that favors bacterial persistence.

Several limitations should be acknowledged. First, we have not yet obtained a high-resolution cryo-EM structure of the PPE57–MmpL3 complex, which we are currently pursuing. Second, due to growth constraints of the H37Rv Δ*PPE57* strain in cholesterol-containing minimal medium, and the technical limitations of performing real-time kinetic assays in BSL-3, some mechanistic experiments were performed using BCG strains. BCG shares conserved lipid transport and cholesterol uptake machinery with *M. tb*, and all key functional conclusions were validated in the virulent background. Third, our in vivo data suggest, but do not prove, a role for PPE57 in latent infection; this needs to be tested using Cornell or reactivation models. Fourth, PPAR-γ dependency was established through pharmacological and expression analyses, but genetic validation using macrophage-specific PPAR-γ knockout is needed for definitive evidence.

In summary, our work establishes PPE57 as an essential nexus connecting mycobacterial cell wall biogenesis with host metabolic exploitation. By physically and functionally coupling with MmpL3, PPE57 ensures efficient mycolic acid transport while simultaneously enabling cholesterol acquisition, which in turn orchestrates host metabolic reprogramming to support persistent infection. These findings illuminate how *M. tb* integrates envelope integrity with nutrient scavenging, thereby revealing PPE57 as a dual-function therapeutic target that disrupts both bacterial cell wall assembly and host metabolic exploitation. This study identifies a vulnerable node in this interface that may be exploitable for host-directed therapy or antivirulence strategies, potentially complementing existing bactericidal drugs.

## Data Availability

The raw RNA sequencing data generated and analyzed in this study are available in the NCBI Sequence Read Archive (SRA) under BioProject accession number PRJNA1490370. The BioProject can be accessed at https://www.ncbi.nlm.nih.gov/bioproject/PRJNA1490370. All other datasets generated and/or analyzed during the current study are included in the article or Supplementary Materials. Further inquiries can be directed to the corresponding author.

## Supplementary Materials

The following supporting information can be downloaded at: https://www.mdpi.com/article/10.3390/microorganisms14091912/s1, Figure S1: Verification of PPE57 deletion. Figure S2: Bacterial permeability detection. Figure S3: The absence of PPE57 leads to changes in the bacterial transcribe landscape. Figure S4: Purification of bacterial protein. Figure S5: Interaction analysis of PPE57 and MmpL3. Figure S6: PPE57 promotes cholesterol uptake in macrophages. Figure S7: PPE57 affects the bacteria’s ability to persist in cells. Table S1: Primers for knockout strain construction. Table S2: Plasmid construction primers.
