# Supplementary material for: PPE57 Cooperates with MmpL3 to Mediate Bacterial Lipid Transport and Promote Persistent Infection of Mycobacterium tuberculosis
Source: Microorganisms. 2026 Aug 28;14(9):1912. doi: 10.3390/microorganisms14091912 (PMC13609142; doi:10.3390/microorganisms14091912)
Supplement: Supplementary file 1 [file microorganisms-14-01912-s001.zip › Supplementary Figures.pdf]

Figure. S1

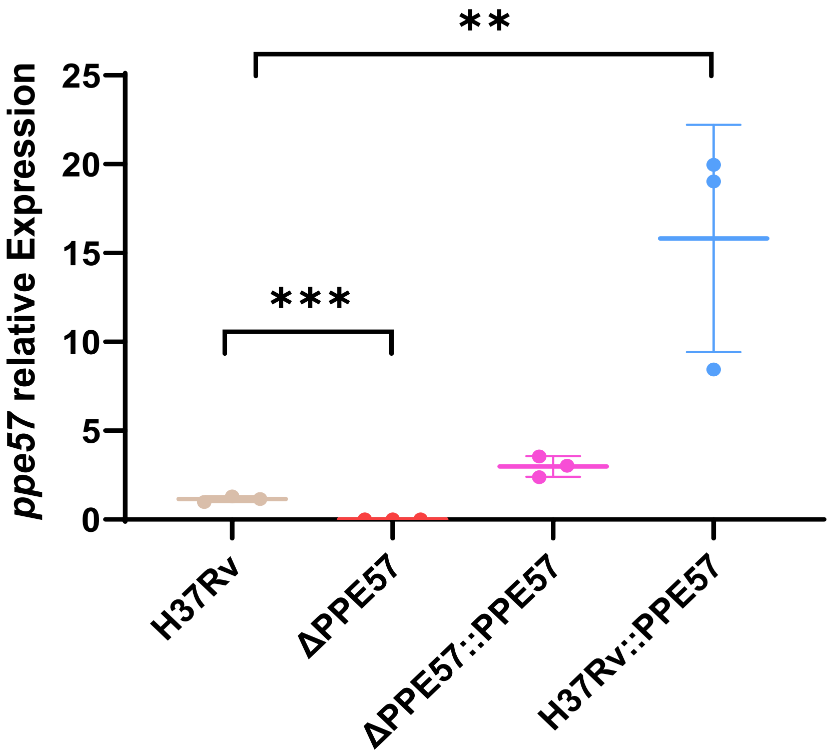

Figure. S1 Verification of PPE57 deletion. Reverse Transcription quantitative PCR(RT-qPCR) was performed to validate *ppe57* gene expression levels. Data are shown as mean ±SD, n = 3. Statistical analysis was performed using one-way ANOVA (**, P < 0.01, ***, P < 0.001).

Figure. S2

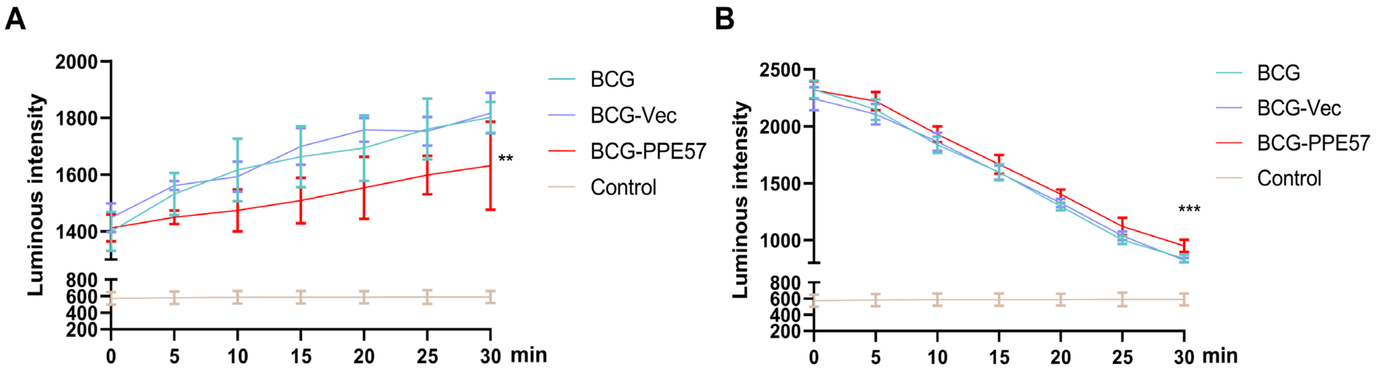

Figure. S2 Bacterial permeability detection. (**A**) Uptake capacity of EB by *BCG*, *BCG-Vec*, and *BCG-PPE57* strains. (**B**) Efflux capacity of EB by *BCG*, *BCG-Vec*, and *BCG-PPE57* strains. Data are shown as mean ±SD, n = 5. Statistical analysis was performed using one-way ANOVA (**, P < 0.01, ***, P < 0.001).

Figure. S3

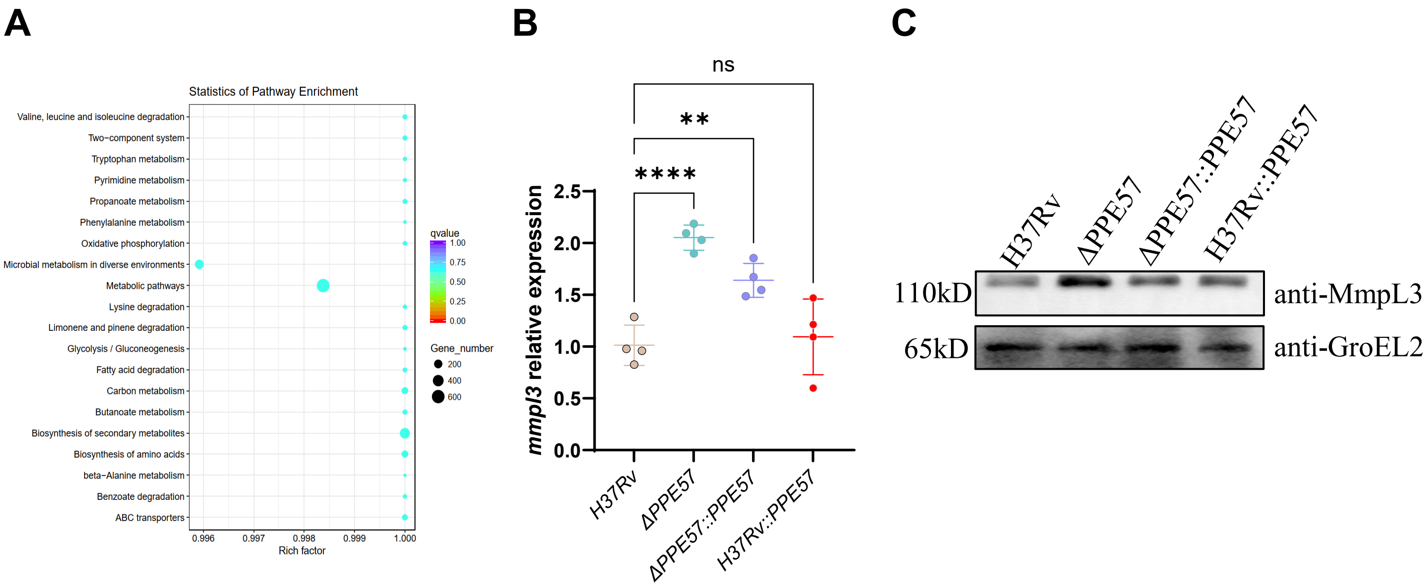

Figure. S3 The absence of PPE57 leads to changes in the bacterial transcribe landscape. (**A**) KEGG pathway analysis of DEGs between *H37Rv* and Δ*PPE57*. (**B**) *mmpl3* gene expression levels of bacteria.(**C**) MmpL3 expression levels of *M. tb*. Data are shown as mean ±SD, n = 4. Statistical analysis was performed using one-way ANOVA (ns, No significance; **, $P < 0.01$, ****, $P < 0.0001$).

Figure. S4

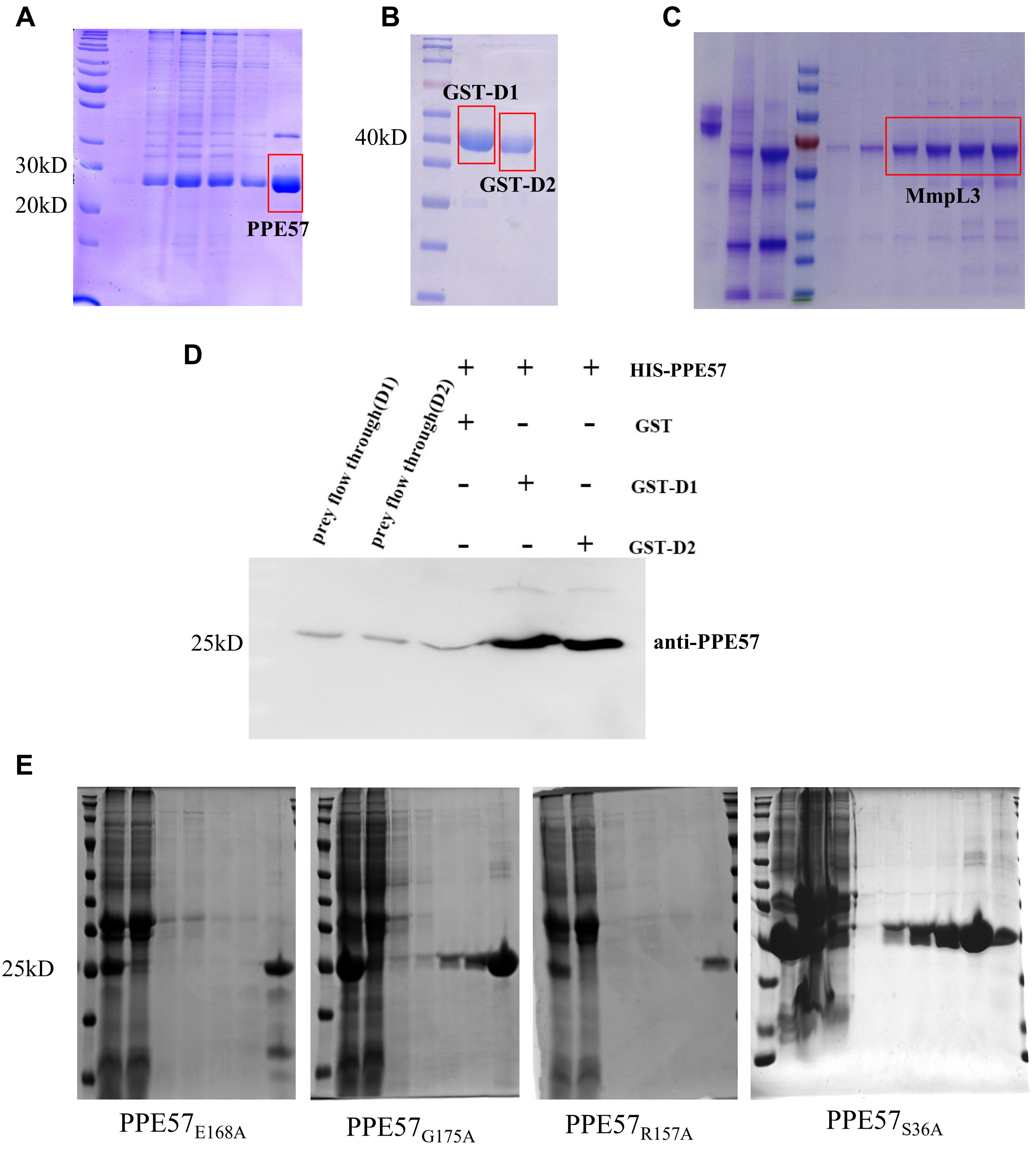

Figure. S4 Purification of bacterial protein. (**A**) Purification of PPE57 protein. (**B**). MmpL3 –GST-D1/GST-D2 protein purification. (**C**) Purification of MmpL3 protein. (**D**) Pull down validation of PPE57 and MmpL3 extracellular domain D1/D2.The purified GST protein hanging column was used as a negative control. (**E**) Purification of PPE57 corresponding mutant protein.

Figure. S5

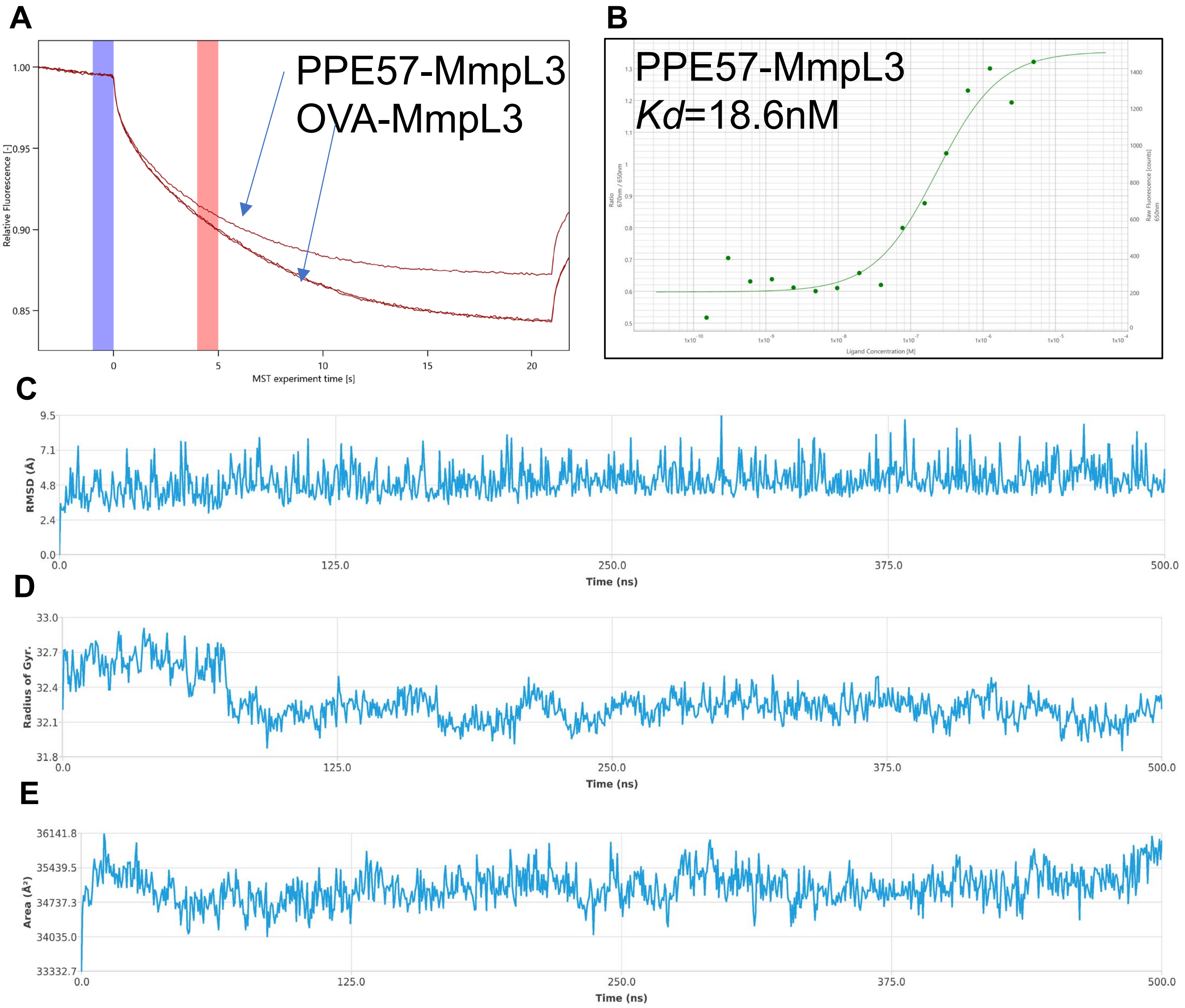

Figure. S5 Interaction analysis of PPE57 and MmpL3. (**A**) MST interaction of PPE57 and MmpL3. (**B**) Determination of binding constants of PPE57 and MmpL3.(**C-E**)Molecular dynamics simulation of PPE57 and MmpL3. **C**: RMSD generally refers to the structural deviation of a certain moment relative to the reference conformation; **D**: RG is an index to measure the mass-weighted average radius of the system during the simulation process; **E**: MSA: Molecular surface area.

Figure. S6

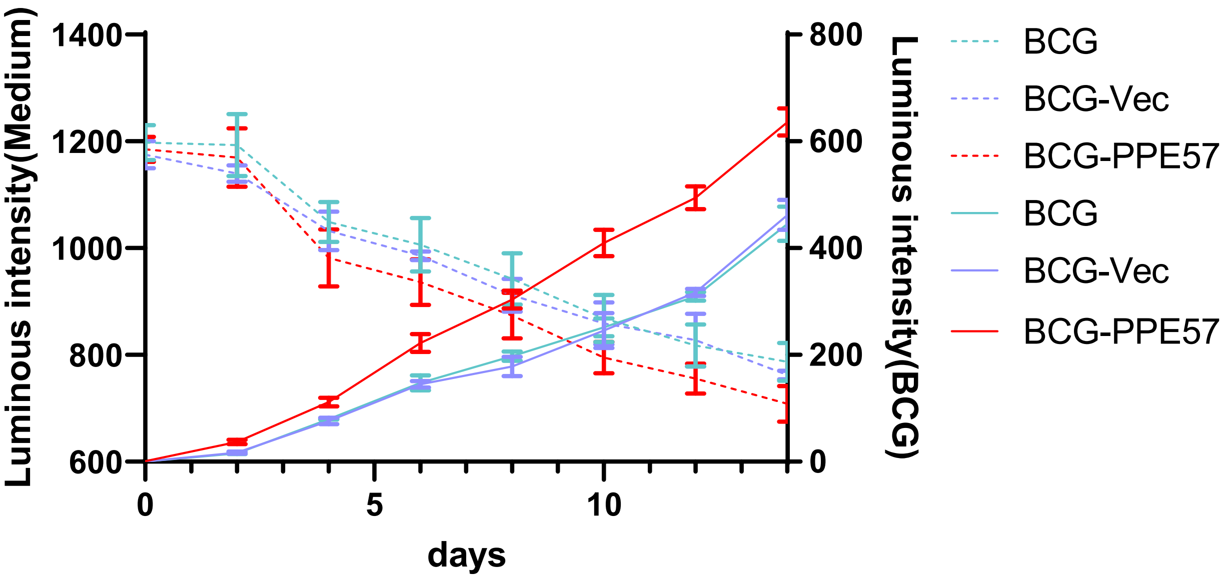

Figure. S6 PPE57 promotes cholesterol uptake in macrophages. Uptake of NBD-cholesterol by *BCG* strains. Log-phase growing bacteria were collected and adjusted to a uniform concentration. They were then cultured in 7H9-OADC medium with the addition of 2 μM NBD-cholesterol (Cat#E15249, Efebio, China). The cultures were incubated statically in the dark at 37℃. At the predetermined time points, 1 mL of the corresponding bacterial suspension was taken out and centrifuged at 8000 rpm. The bacterial pellet was resuspended in PBS. The fluorescence values of the culture supernatant (dashed lines) and the resuspended bacterial pellet(solid lines) were measured using a multifunctional microplate reader.

Figure. S7

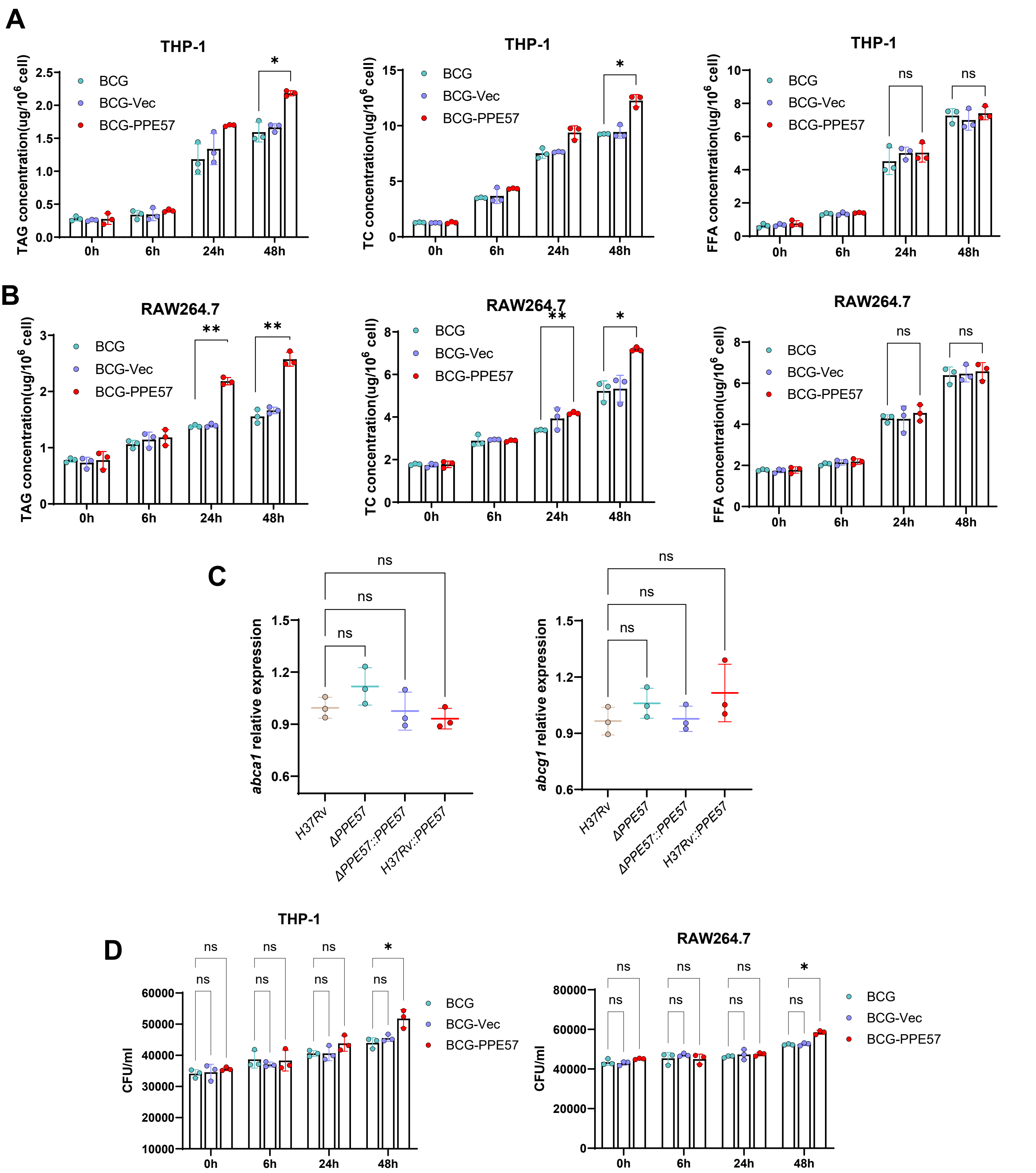

Figure. S7 PPE57 affects the bacteria's ability to persist in cells. (**A**) TAG content, TC content and FFA content in cells infected with BCG strains after infection of THP-1 cells. (**B**) TAG content, TC content and FFA content in cells infected with *BCG* strains after infection of RAW264.7 cells. (**C**) Expression levels of cholesterol efflux genes in THP-1 cells 48 hours after infection with the *H37Rv* strain.(**D**) Bacterial counts of *BCG*, *BCG-Vec*, and *BCG-PPE57* strains infecting THP-1 and RAW264.7 cells. Data are shown as mean ±SD, n = 3. Statistical analysis was performed using one-way ANOVA (ns, No significance; *, $P < 0.05$, **, $P < 0.01$).
