# Supplementary material for: PPE57 Cooperates with MmpL3 to Mediate Bacterial Lipid Transport and Promote Persistent Infection of Mycobacterium tuberculosis
Source: Microorganisms. 2026 Aug 28;14(9):1912. doi: 10.3390/microorganisms14091912 (PMC13609142; doi:10.3390/microorganisms14091912)
Supplement: Supplementary file 1 [file microorganisms-14-01912-s001.zip › Supplementary Table.pdf]

Supplementary Table: Primer sequences used in the construction.

Table S1. Primers for knockout strain construction.

Primer	Sequence
LFP	5'- TTTTTTTT*GCCTAAATGGC*GGTTGGCGTCAATAGAGTGC -3'
LRP	5'- TTTTTTTT*GCCTTTCTGGC*CCGCTGGTATCATTGGATGC -3'
RFP	5'- TTTTTTTT*CCATAGATTGG*CGATCTGCGCTATCGGATCT -3'
RRP	5'- TTTTTTTT*CCATCTTTTGG*CCGGTACTGCTCGTATTGTG -3'

Table S2. Plasmid construction primers.

Plasmid	Primer	Sequence
pMV361-ppe57	F primer	5'-TTGCGGATCCAGCTGCAGAATTCATGCATCCAATGATACC AGCGGAGTAT -3'
	R primer	5'-CTAGTTAACTACGTCGACATCGATAAGCTTTTACCCGCCC CTGTAGATCTGC -3'
pMV261-ppe57	F primer	5'-AATGGATCCATGCATCCAATGATACCAGCGGAGT-3'
	R primer	5'-TTTAAGCTTCCCGCCCCTGTAGATCTGCGGCGGT-3'
